# IGF2BP1/HMOX1 Mediates Fate Determinations of Human Spermatogonial Stem Cells and Male Infertility via an m6A-Dependent Manner

**DOI:** 10.34133/research.1005

**Published:** 2025-11-21

**Authors:** Li Du, Wei Liu, Yinghong Cui, Wei Chen, Zuping He

**Affiliations:** ^1^ Key Laboratory of Model Animals and Stem Cell Biology in Hunan Province, Engineering Research Center of Reproduction and Translational Medicine of Hunan Province, Institute of Interdisciplinary, Hunan Normal University School of Basic Medicine, Changsha 410013, China.; ^2^ Department of Critical Medicine, Hunan Aerospace Hospital, Hunan Normal University, Changsha 410205, China.; ^3^ Department of Pharmacy, Third Xiangya Hospital, Central South University, Changsha 410013, China.; ^4^ Key Laboratory of Reproductive Health Diseases Research and Translation, Ministry of Education, NHC Key Laboratory of Tropical Disease Control, Hainan Academy of Medical Sciences, Hainan Medical University School of Life Sciences and Medical Technology, Haikou 571199, China.

## Abstract

The normal development of spermatogonial stem cells (SSCs) is essential for maintaining male fertility. Nevertheless, signaling molecules and pathways regulating the fate decisions of human SSCs remain elusive. We have reported that Opa interacting protein 5 (OIP5) is involved in controlling human SSC self-renewal and apoptosis. Notably, we found that insulin-like growth factor 2 mRNA-binding protein 1 (IGF2BP1) interacted with OIP5 in human SSCs. Our RNA sequencing (RNA-seq) and immunofluorescence–fluorescence in situ hybridization (IF-FISH) identified heme oxygenase 1 (HMOX1) as a downstream target of IGF2BP1 in human SSCs. Methylated RNA immunoprecipitation-binding qPCR (MeRIP-qPCR) and RNA stability assays revealed that IGF2BP1 could bind to *HMOX1* mRNA and enhance its stability and HMOX1 expression level. Functional assays demonstrated that IGF2BP1 or HMOX1 silencing resulted in the decreased expression levels of ferroptosis-associated genes (e.g., *SLC7A11* and *SLC3A2*), the increased intracellular reactive oxygen species (ROS) level and ferrous ion (Fe^2+^) content, and the impaired stemness maintenance of human SSCs. Additionally, we observed that IGF2BP1-mediated HMOX1 stabilization activated system Xc^−^, thereby inhibiting ferroptosis of human SSCs. Furthermore, *IGF2BP1* gene variants were positively correlated with the occurrence of non-obstructive azoospermia (NOA). Collectively, these results imply a critical role of IGF2BP1/*HMOX1* mRNA/system Xc^−^ axis in regulating human SSC ferroptosis, autophagy, and stemness maintenance. This study thus provides novel insights into the regulatory mechanisms of human SSC fate determinations and offers new targets for treating male infertility.

## Introduction

Infertility has become a serious disease that affects human health and population growth, and it is one of the major reasons for negative population growth in the past several years [1]. Compared to the last 45 years, currently the average sperm count has been decreased by 62%, and sperm concentration is reduced by 52% [2], with at least 30 million men experiencing infertility worldwide [3]. One of the common causes of male infertility and subfertility is spermatogenesis disorders. Spermatogenesis is a complex and highly fine-tuned process that comprises the mitosis of spermatogonia, meiosis of spermatocytes, and spermiogenesis of spermatids [4]. Abnormalities in genetic and epigenetic regulation (e.g., noncoding RNA, DNA methylation, and histone modification) at any of these stages lead to the impaired spermatogenesis and male infertility [5,6]. As the initiating cells of spermatogenesis, human spermatogonial stem cells (SSCs) are required for maintaining normal spermatogenesis and ensuring male fertility [7]. Therefore, the in-depth studies of the fate-determining mechanisms of human SSCs are vital for unveiling the pathogenesis of non-obstructive azoospermia (NOA) and developing potential therapeutic strategies. Nevertheless, genes and their signaling pathways controlling the fate decisions of human SSCs remain largely unknown.

We have recently reported that Opa interacting protein 5 (OIP5) plays a critical role in regulating the self-renewal and apoptosis of human SSCs [8]. Through mass spectrometry (MS) analysis and co-immunoprecipitation (Co-IP) assay, we identified an interaction between OIP5 and IGF2BP1 (insulin-like growth factor 2 mRNA-binding protein 1). Using the Human Protein Atlas (HPA) and the Male Health Atlas (MHA) databases, we observed that IGF2BP1 was highly expressed in human SSCs and spermatocytes, and its expression is decreased in NOA patients. Therefore, we hypothesize that IGF2BP1 plays an important role in controlling fate determinations of human SSCs and that its dysfunction may be associated with male infertility. Notably, it has been recently shown that IGF2BP1 regulates the stability of orthodenticle homeobox 2 (*OTX2*) mRNA via an m6A modification to inhibit the differentiation of embryonic stem cells (ESCs) into primordial germ cells (PGCs) by suppressing the expression of transcription factor AP-2γ (TFAP2C) [9]. This study highlights an indispensable role of IGF2BP1 in mediating the development of human germ cells.

IGF2BP1, as an RNA-binding protein (RBP), is involved in multiple kinds of biological processes [10–12]. As an m6A reader protein, IGF2BP1 recognizes and binds to m6A modification sites on mRNAs, thereby controlling cell growth, development, and the progression of various diseases [13,14]. It also serves as a regulator for the development of ESCs and PGCs [15]. In *Drosophila* testes, IGF2BP1 determines the fate of testicular germline stem cells (GSCs) by counteracting endogenous small interfering RNAs (siRNAs) to stabilize the self-renewal factor Unpaired (*Upd*) RNA, thus maintaining the stem cell pool [16]. It has been shown that IGF2BP1 and IGF2BP3 are primarily expressed in spermatogonia in human testis, and IGF2BP1-positive spermatogonia are likely type A spermatogonia [17], suggesting that IGF2BP1 may also regulate human spermatogenesis and spermatogonial development. Conditional knockout of *Igf2bp1* in mouse testes results in the massive loss of male germ cells in the seminiferous tubules and the eventual Sertoli cell-only syndrome (SCOS) phenotype [18]. However, it has been reported that germ cell-specific knockout of *Igf2bp1* does not affect spermatogenesis or fertility in mice [19], a phenotypic discrepancy that may be attributed to differences in the selection of the Cre recombinase system. Notably, a recent study in *Science* highlights that the deletion or mutation of human *AZFa* genes causes male infertility, whereas *AZFa*-knockout mice produce sperm normally [20], which reflects that molecular mechanisms regulating spermatogenesis may be distinct between mice and humans. Therefore, owing to species differences, the specific functions of IGF2BP1 in human spermatogenesis cannot be directly inferred from studies in *Drosophila* or mice. Although these studies provide valuable insights into understanding mammalian spermatogenesis, the roles of IGF2BP1 in different organisms may vary and thus require further investigation. As such, here we conducted the in-depth analyses on the function and molecular mechanism of IGF2BP1 by utilizing xenotransplantation of human SSCs and cellular and molecular assays, including RNA sequencing (RNA-seq), immunofluorescence–fluorescence in situ hybridization, methylated RNA immunoprecipitation-binding quantitative polymerase chain reaction (MeRIP-qPCR), RNA dot blotting, RNA stability and ferroptosis assays, as well as clinical samples (e.g., peripheral blood and testicular tissues from NOA patients). This study is of particular importance because it can provide novel insights into genetic mechanisms underlying human SSC fate decisions and offers therapeutic targets for gene targeting NOA patients with spermatogenesis disorders.

## Results

### Expression and localization of IGF2BP1 in human SSCs and testicular tissues

We have previously found that OIP5 plays a vital role in regulating the self-renewal and apoptosis of human SSCs [8]. Our MS analysis after OIP5 enrichment revealed that IGF2BP1 could interact with OIP5 in human SSCs (Fig. S1A). Our Co-IP assay demonstrated that IGF2BP1 was able to bind to OIP5 in human SSCs (Fig. S1B), while OIP5 could pull down IGF2BP1 in these cells (Fig. S1C). Immunocytochemical staining further showed that both IGF2BP1 and OIP5 were coexpressed in human SSCs (Fig. S1D). Together, these results imply that IGF2BP1 interacts with OIP5 in human SSCs. Therefore, we hypothesized that IGF2BP1 plays an essential role in mediating the fate determinations of human SSCs.

To unveil the expression of IGF2BP1 in testicular tissues, we analyzed public databases HPA and MHA. The HPA database revealed that IGF2BP1 protein was the most highly expressed in testicular tissues (Fig. S2A). The Cancer Genome Atlas (TCGA) data indicated that IGF2BP1 was highly expressed in testicular germ cell tumors (Fig. S2B). The MHA database showed that IGF2BP1 was mainly expressed in SSCs, the differentiating spermatogonia, leptotene spermatocytes, and zygotene spermatocytes (Fig. S2C). Meanwhile, we found that compared with normal testicular tissues, IGF2BP1 was expressed at a lower level in testicular tissues of 3 types of NOA, including idiopathic NOA (iNOA), Klinefelter syndrome (KS), and AZFa-deletion (AZFa-del) (Fig. S2D and E), as well as in maturation arrest at spermatogonia (spg MA) (Fig. S2F and G), maturation arrest at spermatocytes (Spc MA) (Fig. S2F and G), and SCOS (Fig. S2F and G). These data reflect that low expression level of IGF2BP1 may be associated with the occurrence of NOA.

**Fig. 1.**
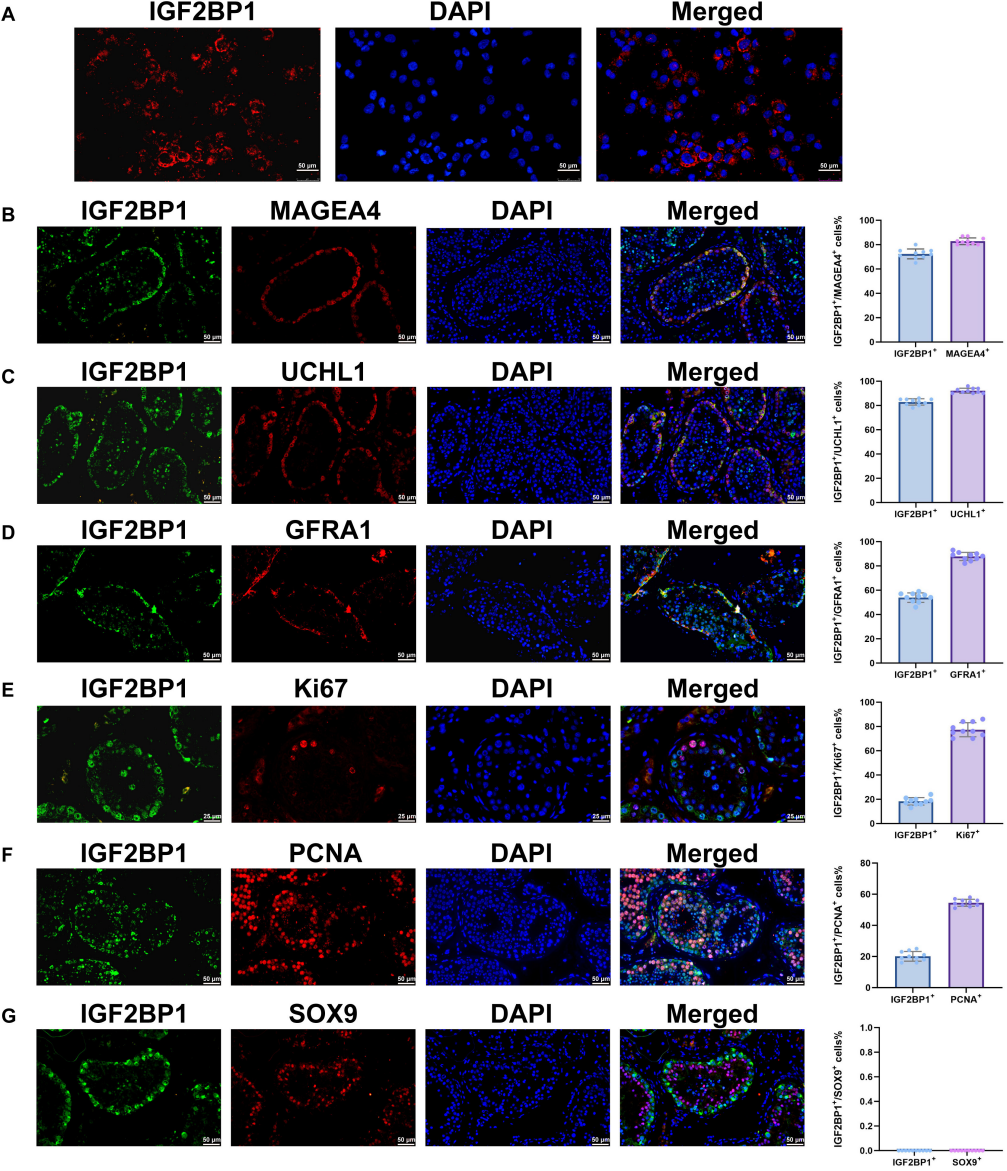
Expression and localization of IGF2BP1 in human SSCs and testicular tissues. (A) Immunocytochemistry showed IGF2BP1 expression in human SSCs. (B) Immunohistochemistry demonstrated colocalization of IGF2BP1 with MAGEA4 in human spermatogonia along the basement membrane of seminiferous tubules of human testes. (C) Immunohistochemistry illustrated colocalization of IGF2BP1 with UCHL1 in human SSCs along the basement membrane of seminiferous tubules of human testes. (D) Double immunostaining indicated colocalization of IGF2BP1 with GFRA1 in human SSCs along the basement membrane of seminiferous tubules of human testes. (E) Immunohistochemistry revealed colocalization of IGF2BP1 with proliferation marker Ki67 in human testicular tissues. (F) Immunohistochemistry showed colocalization of IGF2BP1 with proliferation marker PCNA in human testicular tissues. (G) Immunohistochemistry demonstrated no coexpression of IGF2BP1 with Sertoli cell marker SOX9 in human testicular tissues. Scale bars, 50 μm (A to D, F, and G) and 25 μm (E).

To clarify the expression of IGF2BP1 in human testicular tissues, we performed immunostaining on human SSCs and obstructive azoospermia (OA) patient testicular tissues with normal spermatogenesis. We found that IGF2BP1 was expressed in the cytoplasm of human SSCs (Fig. 1A). Immunohistochemistry illustrated that IGF2BP1 was colocalized with MAGEA4, a marker of human spermatogonia (Fig. 1B), as well as with UCHL1 (Fig. 1C) and GFRA1 (Fig. 1D), hallmarks for human SSCs. These findings suggest that IGF2BP1 may be involved in the stemness maintenance of human SSCs. IGF2BP1 was also colocalized with proliferation markers Ki67 (Fig. 1E) and PCNA (Fig. 1F), indicating its expression in proliferating human SSCs. Our coimmunostaining with SOX9, a marker of human Sertoli cells, revealed no expression of IGF2BP1 in Sertoli cells (Fig. 1G). Collectively, these results implicate that IGF2BP1 is expressed in human SSCs rather than Sertoli cells within testicular tissues.

### IGF2BP1 silencing inhibits the proliferation, self-renewal, DNA synthesis, and stemness maintenance in human SSCs

To investigate the functions and regulatory mechanisms of IGF2BP1 in regulating human SSC fate determinations, we utilized our immortalized human SSC line with long-term proliferation and expansion [21]. We performed biochemical phenotype identification on this human cell line, and we found that it expressed primary human SSC- and spermatogonia-specific genes, including *THY1*, *RET*, *GPR125*, *UCHL1*, *MAGEA4*, and *PLZF* (Fig. S3A). Immunocytochemical staining showed that the human SSC line expressed primary human SSC- and spermatogonia-specific proteins, namely, GFRA1, UCHL1, MAGEA4, PLZF, and GPR125 (Fig. S3B), indicating that this cell line is human SSCs phenotypically. Therefore, this human SSC line can be used to examine the functions and molecular mechanisms of human SSC fate determinations.

**Fig. 2.**
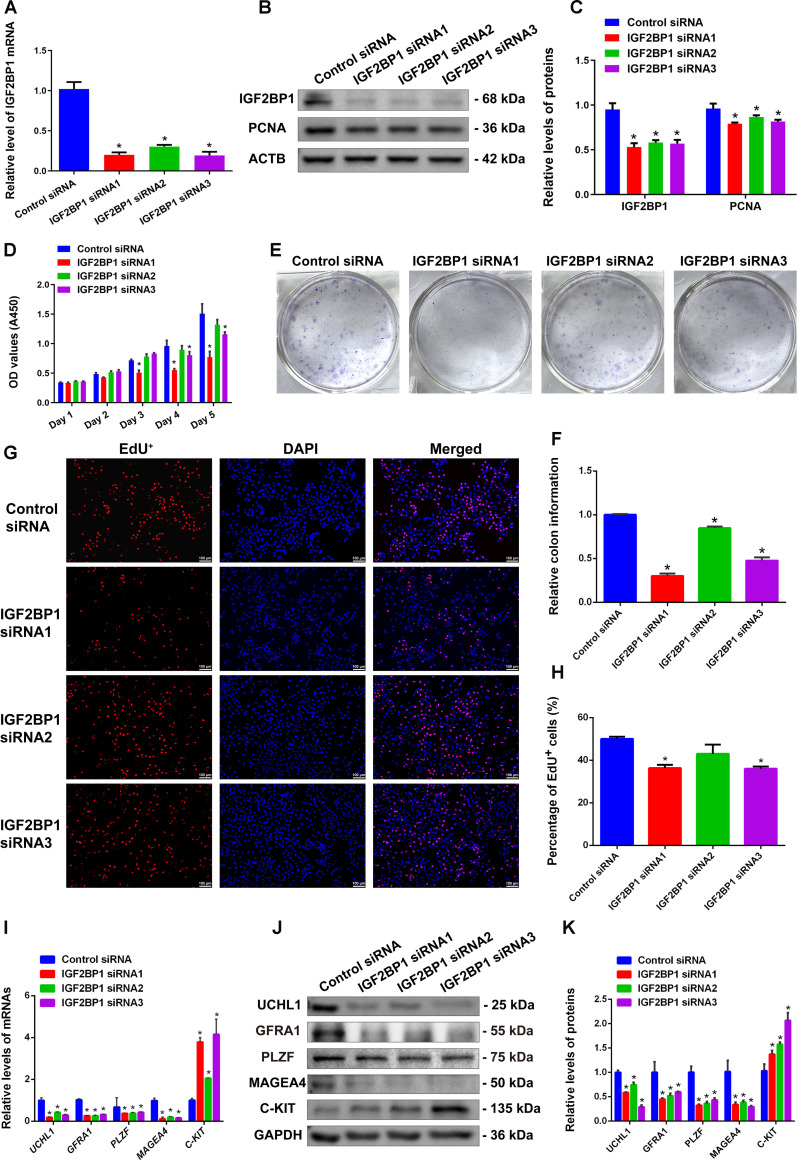
Effect of IGF2BP1 knockdown on the proliferation, DNA synthesis, and stemness maintenance of human SSCs. (A) Real-time PCR showed that IGF2BP1 siRNA1–3 significantly decreased *IGF2BP1* mRNA expression. (B and C) Western blots indicated that IGF2BP1 siRNA1–3 significantly reduced IGF2BP1 and PCNA protein levels. (D) CCK-8 assay demonstrated that IGF2BP1 siRNA1 and siRNA3 significantly inhibited the proliferation of human SSCs. (E and F) Colony formation assay illustrated that IGF2BP1 siRNA1 and siRNA3 significantly suppressed the self-renewal capacity of human SSCs. (G and H) EdU incorporation assays revealed that IGF2BP1 siRNA1 and siRNA3 diminished DNA synthesis in human SSCs. (I to K) Influence of IGF2BP1 knockdown on the mRNA and protein expression levels of UCHL1, GFRA1, PLZF, MAGEA4, and C-KIT in human SSCs. *P < 0.05, with statistically significant differences.

To explore the biological functions of IGF2BP1 in controlling human SSCs, we designed 3 siRNAs targeting *IGF2BP1* to knock down its mRNA and protein expression. Reverse transcription PCR (RT-PCR) and Western blots showed that IGF2BP1 siRNA1, siRNA2, and siRNA3 effectively decreased *IGF2BP1* mRNA and IGF2BP1 protein expression (Fig. 2A to C), with *IGF2BP1* mRNA silencing efficiency exceeding 70%, which reflects that IGF2BP1 siRNA1–3 can be employed for subsequent functional and mechanistic studies of IGF2BP1 in human SSCs. Meanwhile, we found that IGF2BP1 knockdown led to the decrease in the expression level of the proliferating marker PCNA (Fig. 2B and C), suggesting that IGF2BP1 silencing reduces the proliferation of human SSCs. To investigate the role of IGF2BP1 in the proliferation of human SSCs, we performed Cell Counting Kit-8 (CCK-8) assays for 5 consecutive days after transfecting these cells with IGF2BP1 siRNA1–3. Compared to the control siRNA, the absorbance values of human SSCs transfected with IGF2BP1 siRNA1 and siRNA3 showed a significant downward trend (Fig. 2D), indicating that IGF2BP1 knockdown inhibits their proliferation. Additionally, our colony formation assays revealed a notable reduction in the number of colonies formed by human SSCs treated with IGF2BP1 siRNA1 and siRNA3 (Fig. 2E and F), suggesting that IGF2BP1 knockdown impairs the self-renewal of human SSCs. Subsequently, 5-ethynyl-2′-deoxyuridine (EdU) incorporation assays further revealed a notable decrease in the proportion of EdU-positive cells in the IGF2BP1 siRNA-treated human SSCs (Fig. 2G and H), indicating that IGF2BP1 silencing significantly suppresses DNA synthesis in human SSCs. Collectively, these results demonstrate the critical role of IGF2BP1 in regulating DNA synthesis, self-renewal, and the proliferation of human SSCs.

We asked whether IGF2BP1 affects the stemness of human SSCs. Our RT-PCR and Western blots showed that the mRNA and protein expression levels of human SSC markers, including UCHL1, GFRA1, PLZF, and MAGEA4, were significantly decreased by IGF2BP1 silencing in human SSCs (Fig. 2I to K), which implies that the stemness of human SSCs was inhibited by IGF2BP1 knockdown. Subsequently, IGF2BP1 was overexpressed in human SSCs, which resulted in increases in the expression levels of UCHL1, GFRA1, PLZF, and PCNA (Fig. S4A to C), implicating that IGF2BP1 overexpression promotes stemness maintenance and cell proliferation of human SSCs. At the same time, we observed a significant up-regulation in the expression level of the differentiation-related protein c-KIT (Fig. 2I to K), suggesting that IGF2BP1 knockdown promotes cell differentiation. These data indicate the important role of IGF2BP1 in maintaining the stemness of human SSCs and inhibiting their differentiation.

**Fig. 3.**
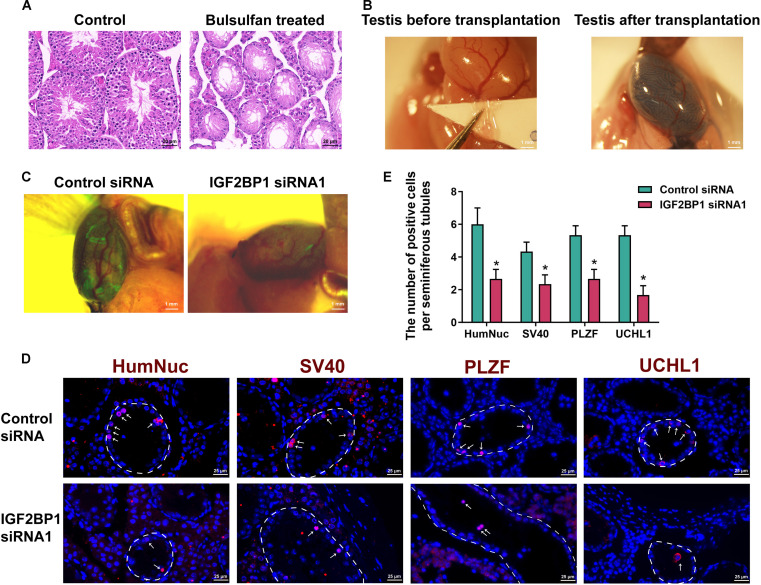
Impact of IGF2BP1 knockdown on self-renewal of human SSCs in vivo*.* (A) H&E staining reflected the successful establishment of the recipient mice without male germ cells (right panel) with treatment of 40 mg/kg body weight busulfan. Scale bars, 20 μm. (B) Recipient testicular morphology of mice before and after xenotransplantation with human SSCs showed seminiferous tubules stained trypan blue (right panel). Scale bars, 1 mm. (C) The fluorescence intensity of GFP in the recipient mouse testes was observed under a stereomicroscope 4 weeks after human SSC transplantation. (D and E) Immunohistochemistry showed the expression of human nuclear antigen (HumNuc), SV40, PLZF, and UCHL1 proteins in recipient mouse testicular seminiferous tubules at 4 weeks after human SSCs’ xenotransplantation. Scale bars, 25 μm. *P < 0.05, with statistically significant differences.

To assess the effect of IGF2BP1 on self-renewal in vivo, we performed xenotransplantation of human SSCs into recipient mouse seminiferous tubules. Busulfan with 40 mg/kg body weight was used to remove male germ cells in the recipient mouse testes. Hematoxylin and eosin (H&E) staining showed that, compared with the control group, the seminiferous tubules of busulfan-treated mice had no male germ cells with only Sertoli cells (Fig. 3A). Subsequently, human SSCs transfected with the control siRNA or IGF2BP1 siRNA1 were transplanted into the seminiferous tubules of recipient mice. Owing to the immune privilege of the testicular tissues [22,23], human SSCs transplanted via the efferent duct were able to colonize in the mouse testes. Human SSCs were mixed with trypan blue at a ratio of 1:1, and they were transplanted into seminiferous tubules of recipient mice via the efferent ducts (Fig. 3B, left). Successfully transplanted testes were shown in Fig. 3B, right. Given that our human SSC line expresses enhanced green fluorescent protein (eGFP), we observed a significant reduction in GFP fluorescence in the IGF2BP1 siRNA1 group compared to the control siRNA at 4 weeks after transplantation under a stereofluorescence microscope (Fig. 3C), suggesting that IGF2BP1 silencing reduces cell colonization of human SSCs. To unequivocally identify the transplanted human SSCs within the mouse testicular environment, their localization was determined by detecting the coexpression of 2 specific proteins. HumNuc, a human nuclear antigen, can distinguish human cells from the host murine tissues. Additionally, SV40 was utilized because the human SSC line employed in this study was immortalized through transfection with the SV40 large T antigen. We then analyzed the survival of human SSCs in mouse testes by immunofluorescence staining for HumNuc, SV40, PLZF, and UCHL1. As shown in Fig. 3D and E, compared with the control siRNA, human SSCs transfected with IGF2BP1 siRNA1 exhibited the reduced colonization in recipient mouse testes, with significantly fewer HumNuc-, SV40-, PLZF-, and UCHL1-positive cells. These results indicate that IGF2BP1 knockdown significantly impairs the in vivo self-renewal capacity of human SSCs.

### IGF2BP1 knockdown enhances the ferroptosis of human SSCs by inhibiting autophagy

**Fig. 4.**
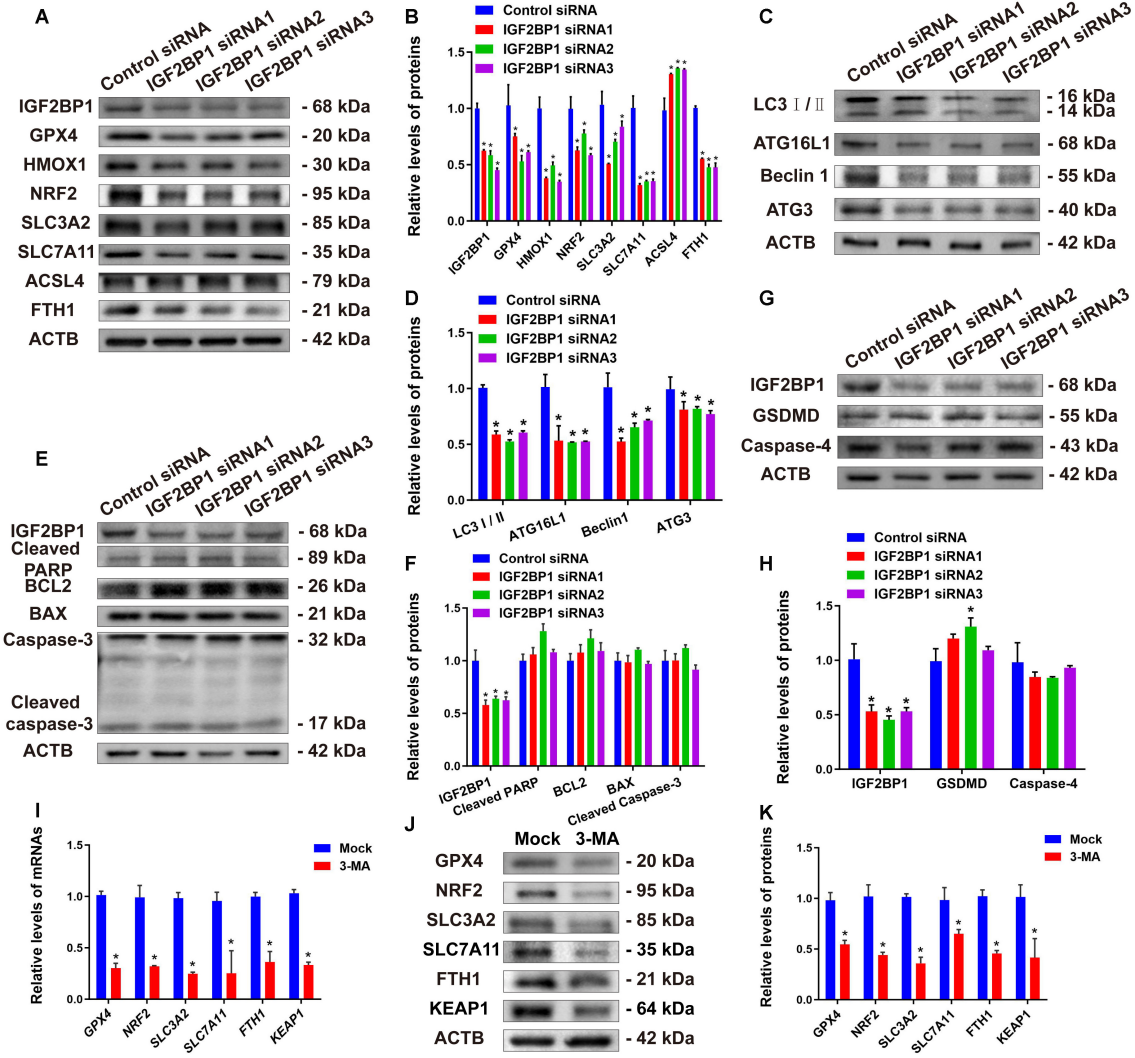
Influence of IGF2BP1 knockdown on several types of cell death in human SSCs. (A and B) Expression levels of ferroptosis marker proteins GPX4, HMOX1, NRF2, SLC3A2, SLC7A11, ACSL4, and FTH1 in human SSCs after IGF2BP1 knockdown. (C and D) Expression levels of autophagy marker proteins LC3I/II, ATG16L1, Beclin1, and ATG3 in human SSCs with the control siRNA and IGF2BP1 siRNA1–3. (E and F) Expression levels of apoptosis marker proteins BCL2, BAX, cleaved PARP, and cleaved caspase-3 in human SSCs after IGF2BP1 knockdown. (G and H) Expression changes in pyroptosis marker proteins GSDMD and caspase-4 in human SSCs by IGF2BP1 silencing. (I to K) Changes in ferroptosis-related genes and proteins GPX4, NRF2, SLC3A2, SLC7A11, FTH1, and KEAP1 in human SSCs after treatment with autophagy inhibitor 3-MA. *P < 0.05, with statistically significant differences.

To further investigate whether IGF2BP1 is involved in regulating various cell death pathways in human SSCs, we analyzed the expression changes of key proteins associated with apoptosis, pyroptosis, ferroptosis, and autophagy by IGF2BP1 silencing. Following IGF2BP1 knockdown, the expression levels of ferroptosis-related proteins HMOX1, NRF2, SLC3A2, SLC7A11, GPX4, and FTH1 were significantly down-regulated in human SSCs (Fig. 4A and B), accompanied by up-regulation of the pro-ferroptotic protein ACSL4. These results suggest that IGF2BP1 silencing promotes ferroptosis in human SSCs. Notably, we observed significant decreases in the expression levels of autophagy-related proteins LC3I/II, ATG16L1, Beclin1, and ATG3 by IGF2BP1 knockdown in human SSCs (Fig. 4C and D), indicating that IGF2BP1 silencing reduces autophagy in human SSCs. Additionally, we found no significant changes in the expression levels of proteins associated with the classical apoptotic pathway, e.g., BCL2, BAX, cleaved poly(adenosine diphosphate-ribose) polymerase (PARP), and cleaved caspase-3 (Fig. 4E and F), or pyroptosis-related proteins GSDMD and caspase-4 (Fig. 4G and H) in human SSCs by IGF2BP1 knockdown, suggesting that IGF2BP1 does not affect the apoptosis or pyroptosis of human SSCs. Collectively, these results indicate that IGF2BP1 regulates ferroptosis and autophagy but not apoptosis or pyroptosis in human SSCs. To further demonstrate whether there is an interaction between autophagy and ferroptosis affected by IGF2BP1 in human SSCs, we employed 3-methyladenine (3-MA), a classical autophagy inhibitor, to intervene in human SSCs and explore the specific effect of autophagy regulation on ferroptosis. Interestingly, we found that gene and protein expression levels of ferroptosis markers, including GPX4, NRF2, SLC3A2, SLC7A11, FTH1, and KEAP1, were significantly decreased in human SSCs treated with 3-MA (Fig. 4I to K). These results indicate that the autophagy inhibitor enhances the ferroptosis of human SSCs and that IGF2BP1 regulates the ferroptosis by modulating autophagic activity of human SSCs.

**Fig. 5.**
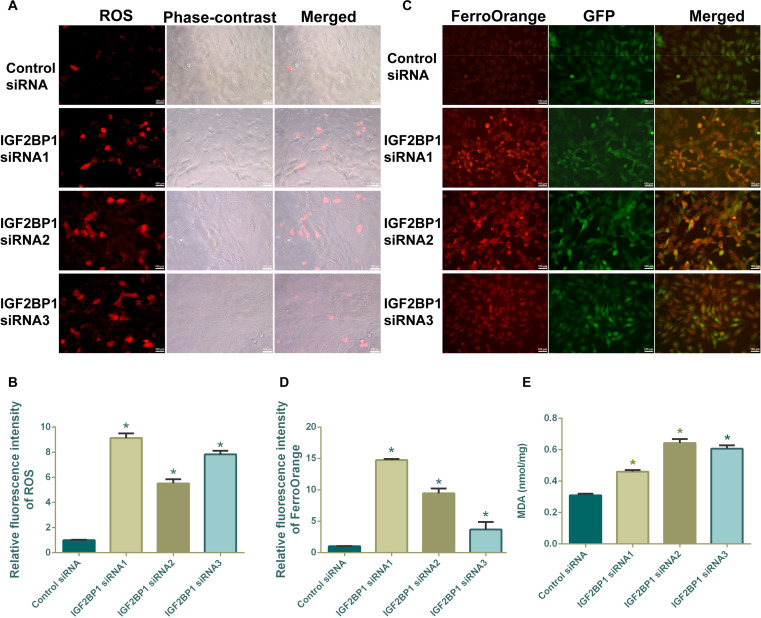
IGF2BP1 knockdown increased the ferroptosis of human SSCs. (A and B) Fluorescence imaging showed the increases in ROS levels of human SSCs after transfection with IGF2BP1 siRNA1–3. (C and D) FerroOrange fluorescence imaging revealed the enhancement in ferrous ion (Fe^2+^) levels in human SSCs after transfection with IGF2BP1 siRNA1–3. (E) MDA content in human SSCs after transfection with IGF2BP1 siRNA1–3 detected by MDA assay kit. *P < 0.05, with statistically significant differences. Scale bars, 100 μm.

To demonstrate whether IGF2BP1 knockdown induces ferroptosis in human SSCs, we comprehensively evaluated ferroptosis-related markers by detecting intracellular reactive oxygen species (ROS), ferrous ion (Fe^2+^) content, and lipid peroxidation levels. We used the dihydroethidium (DHE) fluorescent probe to label intracellular ROS, and we found that IGF2BP1 knockdown significantly enhanced DHE fluorescence intensity in human SSCs (Fig. 5A and B), indicating the increased intracellular oxidative stress. FerroOrange fluorescent probe assays showed that Fe^2+^ levels were significantly elevated in human SSCs transfected with IGF2BP1 siRNA1–3 (Fig. 5C and D), implicating that IGF2BP1 knockdown disrupts intracellular iron homeostasis. Additionally, malondialdehyde (MDA) kit assays revealed a significant increase in MDA content in human SSCs by IGF2BP1 knockdown (Fig. 5E), reflecting the enhanced membrane lipid peroxidation. Collectively, these results indicate that IGF2BP1 knockdown induces ferroptosis in human SSCs by elevating the ROS accumulation, Fe^2+^ overload, and lipid peroxidative damage. Furthermore, we found that IGF2BP1 overexpression significantly up-regulated the expression levels of ferroptosis-related genes and proteins GPX4, NRF2, SLC3A2, SLC7A11, and FTH1 in human SSCs (Fig. S4D to F). Additionally, as a downstream target of IGF2BP1, the expression level of HMOX1 transcript and protein was also markedly increased (Fig. S4D to F), which further indicates that IGF2BP1 affects the occurrence of ferroptosis in human SSCs by regulating the expression of ferroptosis marker proteins.

### HMOX1 is a target of IGF2BP1 in human SSCs

**Fig. 6.**
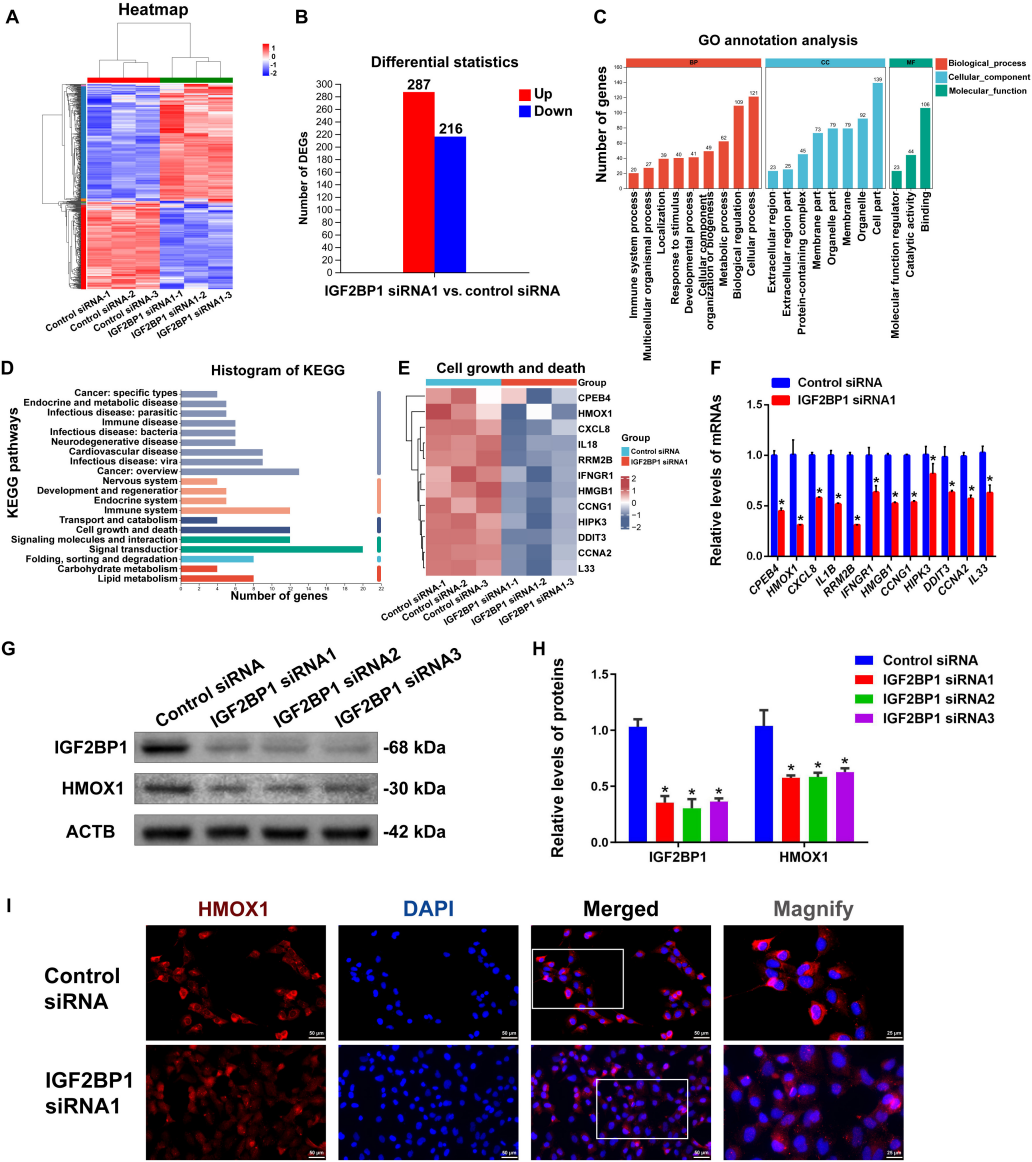
HMOX1 was a target for IGF2BP1 in human SSCs. (A) Gene expression clustering analysis illustrated the global impact of IGF2BP1 silencing on the transcriptome of human SSCs, where red indicated the up-regulated genes and blue denoted the down-regulated genes. (B) Bar chart showed that IGF2BP1 silencing led to 287 up-regulated genes and 216 down-regulated genes in human SSCs. (C) GO analysis of down-regulated DEGs in human SSCs by IGF2BP1 silencing. (D) KEGG analysis of down-regulated DEGs in human SSCs by IGF2BP1 silencing. (E) Clustering analysis of DEGs in human SSCs for growth and death pathways. (F) Real-time PCR showed DEGs in human SSCs by IGF2BP1 silencing. (G and H) Western blot revealed changes in HMOX1 and IGF2BP1 protein expression after transfection with IGF2BP1 siRNA1–3 in human SSCs. (I) Immunocytochemistry showed the changes in HMOX1 fluorescence intensity and localization in human SSCs after IGF2BP1 knockdown. *P < 0.05, with statistically significant differences. Scale bars, 50 μm.

To identify the targets of IGF2BP1 in regulating the fate determinations of human SSCs, we used RNA-seq to comprehensively analyze the transcriptomic changes in human SSCs after IGF2BP1 knockdown. Differentially expressed genes (DEGs) were screened using DESeq2 software with strict criteria: |log₂FC| ≥ 0.585 and *P* < 0.05. We observed 503 DEGs, including 216 down-regulated genes and 287 up-regulated genes, in human SSCs by IGF2BP1 knockdown (Fig. 6A and B). Subsequently, functional enrichment analysis was performed on the down-regulated DEGs. Gene Ontology (GO) analysis revealed that the down-regulated DEGs were primarily enriched in cellular process, biological regulation, binding, and catalytic activity (Fig. 6C), indicating that IGF2BP1, as an RBP, may exert biological functions through binding to other molecules and catalyzing the related biochemical reactions. Further Kyoto Encyclopedia of Genes and Genomes (KEGG) analysis revealed that IGF2BP1 was primarily involved in key pathways, including signal transduction, signal molecule interaction, cell growth, and death (Fig. 6D), suggesting that IGF2BP1 plays a crucial role in regulating cell development and death. We then conducted a heatmap analysis of 12 DEGs related to cell growth and death (Fig. 6E), and validated their expression by real-time PCR showing that *HMOX1* and *RRM2B* were significantly down-regulated in human SSCs by IGF2BP1 silencing (Fig. 6F). Further KEGG analysis indicated that HMOX1 plays a regulatory role in the ferroptosis pathway associated with cell growth and death. Notably, IGF2BP1 siRNA1–3 significantly reduced HMOX1 protein expression (Fig. 6G and H) and altered its subcellular localization with a marked decrease in cytoplasmic localization (Fig. 6I). Therefore, we identified HMOX1 as a downstream target of IGF2BP1 in human SSCs.

### IGF2BP1 interacts with *HMOX1* mRNA in human SSCs

It has recently been reported that IGF2BP1 regulates the stability of *OTX2* mRNA in an m6A-dependent manner to control the differentiation of human ESCs into PGCs [9]. IGF2BP1 can also affect the stability of *ITGB1* mRNA through m6A modification to promote its translation and facilitate cancer cell metastasis and drug resistance [24]. Based on these findings, we hypothesize that IGF2BP1 regulates the proliferation, stemness maintenance, and ferroptosis of human SSCs by influencing the stability or translation of specific genes via an m6A manner.

**Fig. 7.**
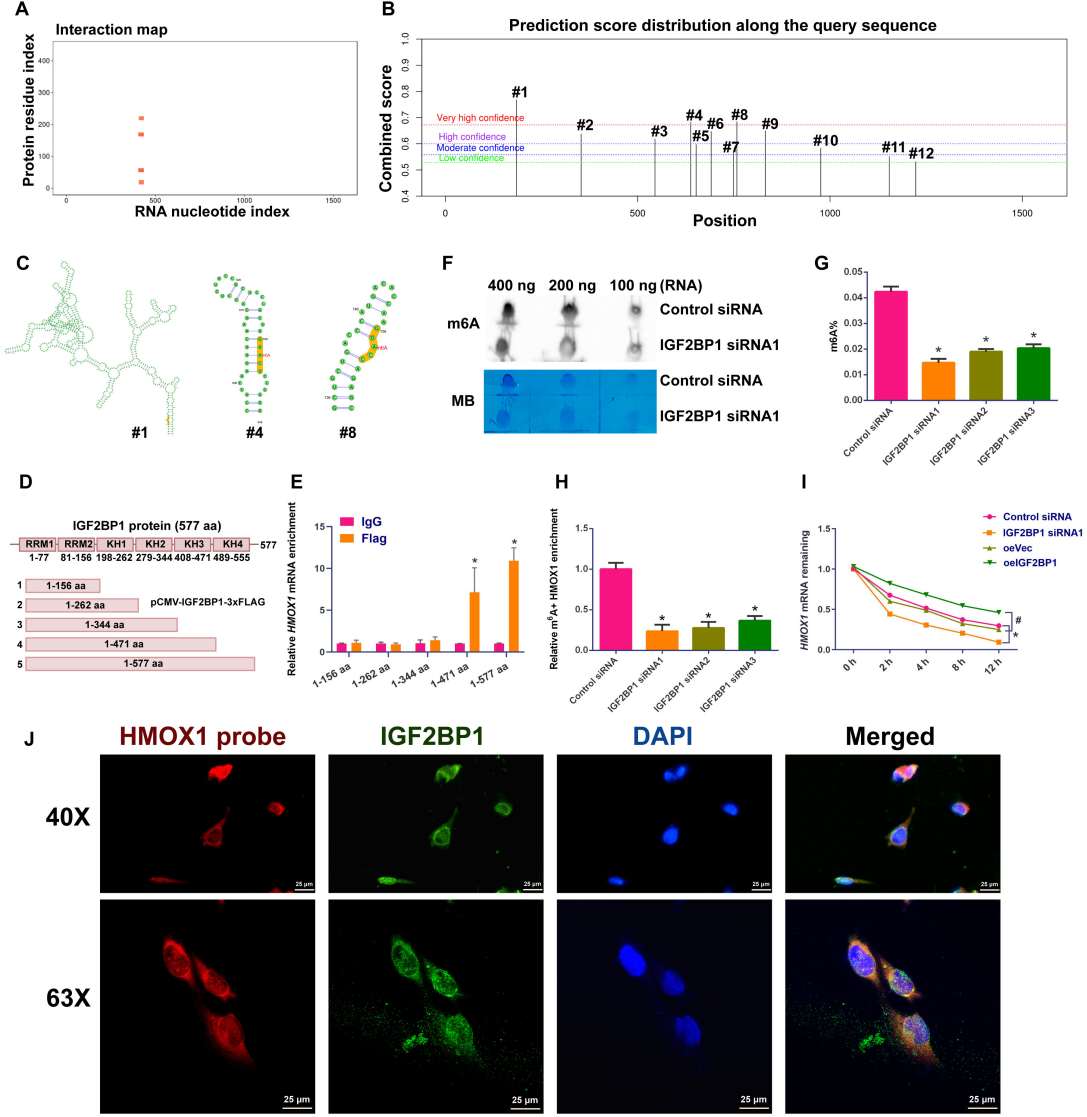
Interaction between IGF2BP1 protein and *HMOX1* mRNA in human SSCs. (A) The catRAPID online tool predicted an interaction between IGF2BP1 protein and *HMOX1* mRNA. (B) The SRAMP website indicated potential m6A modification sites in *HMOX1* mRNA. (C) Secondary structure and specific positions of high-confidence m6A modification sites. (D) Schematic representation of the full-length IGF2BP1 protein and its various deletion mutants used in the RNA immunoprecipitation (RIP) assay. (E) RIP-qPCR analysis of *HMOX1* mRNA enrichment in human SSCs expressing the indicated mutants. (F) RNA dot blotting detected changes in global m6A modification levels in human SSCs. (G) The m6A RNA methylation quantification assay measured the changes in global m6A modification in human SSCs. (H) MeRIP-qPCR detected the changes in m6A-modified *HMOX1* mRNA levels in human SSCs after IGF2BP1 knockdown. (I) The mRNA stability assay measured the decay rate of *HMOX1* mRNA in human SSCs after IGF2BP1 knockdown or overexpression. (J) Confocal microscopy with IF-FISH illustrated the colocalization of *HMOX1* mRNA and IGF2BP1 protein in human SSCs. Scale bars, 25 μm. *P < 0.05, with statistically significant differences.

To explore whether IGF2BP1 can directly interact with *HMOX1* mRNA, we utilized the catRAPID online tool (http://s.tartaglialab.com/page/catrapid_group) to predict the interaction between IGF2BP1 protein and *HMOX1* mRNA. Interestingly, we found strong interaction signals between IGF2BP1 protein and *HMOX1* mRNA (Fig. 7A), which reflects that IGF2BP1 may regulate *HMOX1* mRNA stability or translation efficiency by its direct binding. To investigate whether IGF2BP1 binding to HMOX1 mRNA depends on m6A modification, we used the SRAMP online tool (http://www.cuilab.cn/sramp) to seek potential m6A modification sites in *HMOX1* mRNA. We identified 12 potential m6A modification sites in *HMOX1* mRNA (Fig. 7B). Subsequently, we analyzed the distribution of these high-confidence m6A modification sites in the *HMOX1* mRNA structure, illustrated the secondary structure of *HMOX1* mRNA, and focused on the spatial positions of sites 1, 4, and 8 (Fig. 7C). It has been demonstrated that IGF2BP1 primarily recognizes m6A-modified RNA sequences through its KH3 and KH4 domains [25]. To map the interaction domain between IGF2BP1 and *HMOX1* mRNA, we performed an in vitro binding assay with truncated IGF2BP1 protein. Deletion of the KH3 and KH4 domains (405 to 553 amino acids) in the FLAG-tagged IGF2BP1 protein abrogated its binding to *HMOX1* mRNA (Fig. 7D and E). Taken together, these results indicate that IGF2BP1 may act via binding to *HMOX1* mRNA in an m6A modification-dependent manner. To investigate the effect of IGF2BP1 on m6A modification levels in human SSCs, we employed dot blotting to detect changes in global m6A modification. We observed that IGF2BP1 knockdown significantly reduced the global m6A modification levels in human SSCs (Fig. 7F). Quantitative detection of m6A RNA methylation further displayed that IGF2BP1 silencing led to a notable decrease in m6A modification levels (Fig. 7G), which was consistent with our dot blotting result and supports the critical role of IGF2BP1 in regulating m6A modification in human SSCs. Subsequently, our MeRIP-qPCR analysis revealed that IGF2BP1 knockdown significantly decreased the m6A-modified RNA levels of *HMOX1* mRNA (Fig. 7H), indicating that IGF2BP1 may influence the stability and expression of *HMOX1* mRNA by regulating its m6A modification. RNA stability assays further showed that knockdown of IGF2BP1 accelerated the decay rate of *HMOX1* mRNA in human SSCs (Fig. 7I) and significantly shortened its half-life. Conversely, overexpression of IGF2BP1 slowed the decay rate of *HMOX1* mRNA (Fig. 7I). Furthermore, we determined the colocalization of IGF2BP1 protein and *HMOX1* mRNA, and notably, our IF-FISH assay demonstrated that *HMOX1* mRNA was coexpressing with IGF2BP1 in human SSCs (Fig. 7J), indicating that IGF2BP1 regulates human SSCs by binding to *HMOX1* mRNA. Collectively, these findings suggest that IGF2BP1 enhances the stability of *HMOX1* mRNA by promoting its m6A modification in human SSCs.

### HMOX1 knockdown inhibits the proliferation, self-renewal, and DNA synthesis of human SSCs

Next, we asked whether HMOX1 is involved in regulating the fate determinations of human SSCs. We applied double immunostaining to detect the expression of HMOX1 in human testicular tissues and human SSCs. In normal human testicular tissues, HMOX1 was found to coexpress with the spermatogonial marker MAGEA4 (Fig. S5A) and the SSC marker protein UCHL1 (Fig. S5B). Immunocytochemistry showed that HMOX1 was colocalized with IGF2BP1 in human SSCs (Fig. S5C), reflecting a potential interaction between HMOX1 and IGF2BP1 in these cells. Moreover, HMOX1 was coexpressing with the SSC marker proteins UCHL1 (Fig. S5D) and GFRA1 (Fig. S5E), suggesting that HMOX1 might be involved in the fate determinations of human SSCs.

**Fig. 8.**
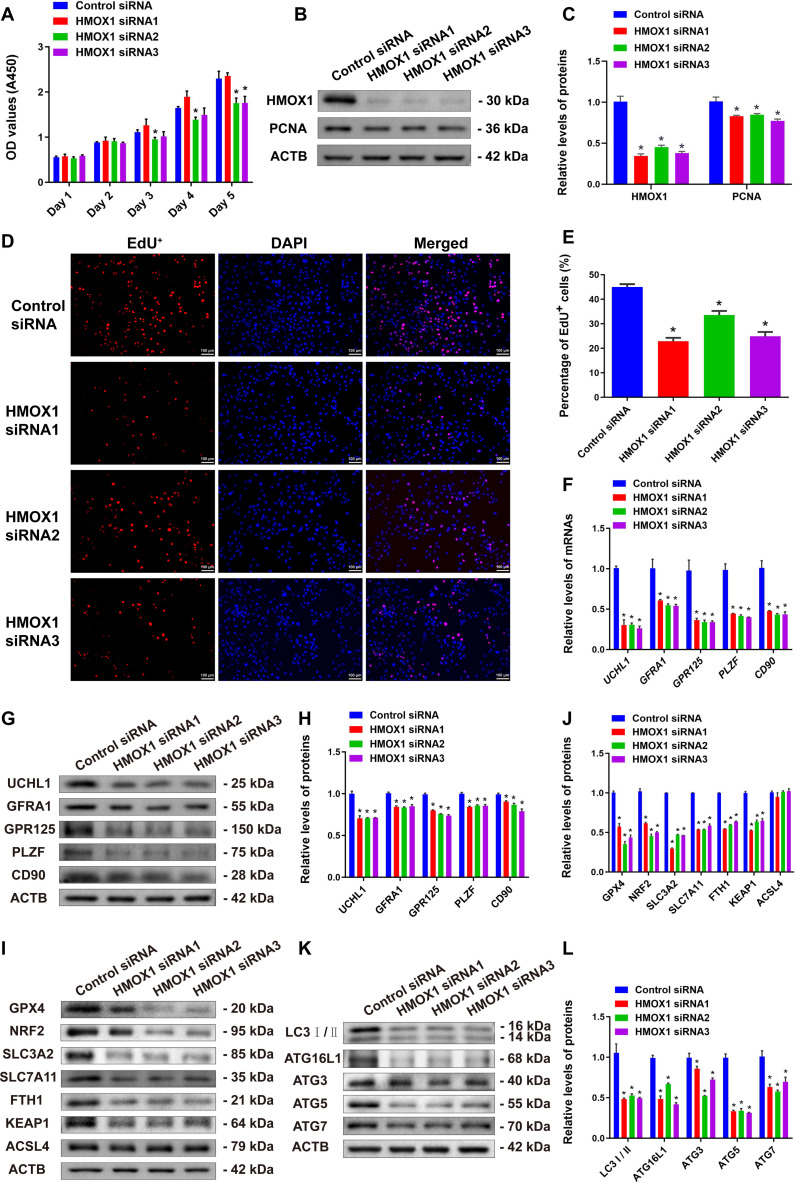
Effect of HMOX1 knockdown on fate decisions of human SSCs. (A) CCK-8 assay detected the effect of HMOX1 knockdown on the proliferation of human SSCs. (B and C) Western blot evaluated the influence of HMOX1 knockdown on PCNA protein levels of human SSCs. (D and E) EdU incorporation assay assessed the impact of HMOX1 knockdown on DNA synthesis of human SSCs. Scale bars, 100 μm. (F) Real-time PCR detected expression changes in SSC marker genes in human SSCs after HMOX1 knockdown. (G and H) Western blot analyzed changes in SSC marker proteins in human SSCs after HMOX1 knockdown. (I and J) Western blot detected changes in ferroptosis-related protein expression in human SSCs after HMOX1 knockdown. (K and L) Western blot revealed changes in autophagy-related protein expression in human SSCs after HMOX1 knockdown. *P < 0.05, with statistically significant differences

After knockdown of HMOX1 in human SSCs, we performed CCK-8 assays, Western blotting, and EdU incorporation assays to evaluate its impact on human SSC proliferation. CCK-8 assays showed that human SSC proliferation was significantly inhibited by HMOX1 siRNA1–3 (Fig. 8A). Western blot analysis revealed a remarkable decrease in the expression of PCNA in human SSCs by HMOX1 knockdown (Fig. 8B and C). EdU incorporation assays further demonstrated a significant reduction in the number of EdU-positive cells in human SSCs by HMOX1 silencing (Fig. 8D and E). Together, these findings suggest that HMOX1 plays an important role in regulating human SSC proliferation and DNA synthesis. Moreover, we observed that knockdown of HMOX1 led to significant down-regulation of both mRNA and protein levels of key stemness markers for SSCs, including UCHL1, GFRA1, GPR125, PLZF, and CD90 (also known as THY1) (Fig. 8F to H). Combining these results with our previous findings, we hypothesize that IGF2BP1 may regulate the expression of HMOX1, thereby influencing stemness maintenance of human SSCs.

To investigate the potential role of HMOX1 in controlling the autophagy and ferroptosis of human SSCs, we detected changes in key proteins related to autophagy and ferroptosis after HMOX1 knockdown. Western blots showed that HMOX1 knockdown significantly reduced the protein expression levels of GPX4, NRF2, SLC3A2, SLC7A11, FTH1, and KEAP1 in human SSCs (Fig. 8I and J), suggesting its involvement in the occurrence of ferroptosis. HMOX1 knockdown also led to the decreases of autophagy-related proteins ATG16L1, ATG3, ATG5, and ATG7 (Fig. 8K and L) but did not affect the expression of apoptosis- or pyroptosis-related proteins (Fig. S6A and B).

**Fig. 9.**
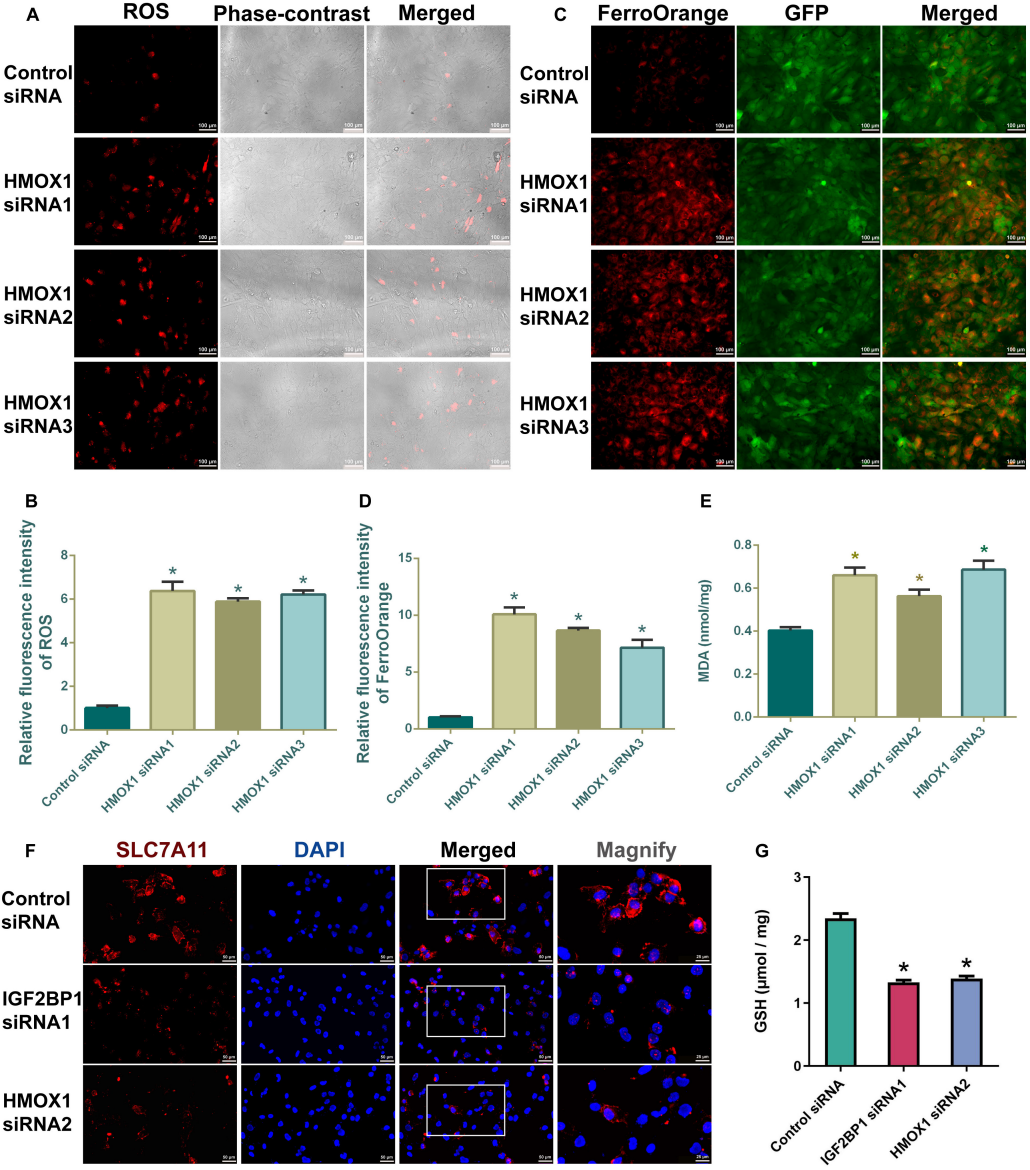
HMOX1 silencing increased the ferroptosis in human SSCs. (A and B) Fluorescence imaging showed the increases in ROS levels of human SSCs after transfection with HMOX1 siRNA1–3. Scale bars, 100 μm. (C and D) FerroOrange fluorescence imaging revealed enhancement in ferrous ion (Fe^2+^) levels of human SSCs after transfection with HMOX1 siRNA1–3. Scale bars, 100 μm. (E) MDA content in human SSCs after transfection with HMOX1 siRNA1–3 detected by MDA assay kit. (F) Immunofluorescence showed the effect of IGF2BP1 and HMOX1 knockdown on the expression and cellular localization of SLC7A11 in human SSCs. Scale bars, 25 and 50 μm. (G) GSH synthesis was decreased in human SSCs by IGF2BP1 and HMOX1 knockdown. *P < 0.05, with statistically significant differences.

We further detected intracellular ROS content, Fe^2+^ concentration, and MDA in human SSCs affected by HMOX1 knockdown. The level of lipid peroxidation in human SSCs was significantly increased by HMOX1 siRNA1–3 (Fig. 9A and B), while the intracellular free Fe^2+^ was elevated in human SSCs by HMOX1 siRNA1–3 (Fig. 9C and D). The MDA content was significantly enhanced in human SSCs by HMOX1 silencing (Fig. 9E), whereas the content of glutathione (GSH) in human SSCs was decreased by HMOX1 silencing (Fig. 9G). Collectively, these data imply that HMOX1 plays a role in inhibiting ferroptosis in human SSCs by regulating the balance of iron metabolism and antioxidant capacity.

### Overexpression of HMOX1 reverses the effect of IGF2BP1 knockdown on human SSCs

**Fig. 10.**
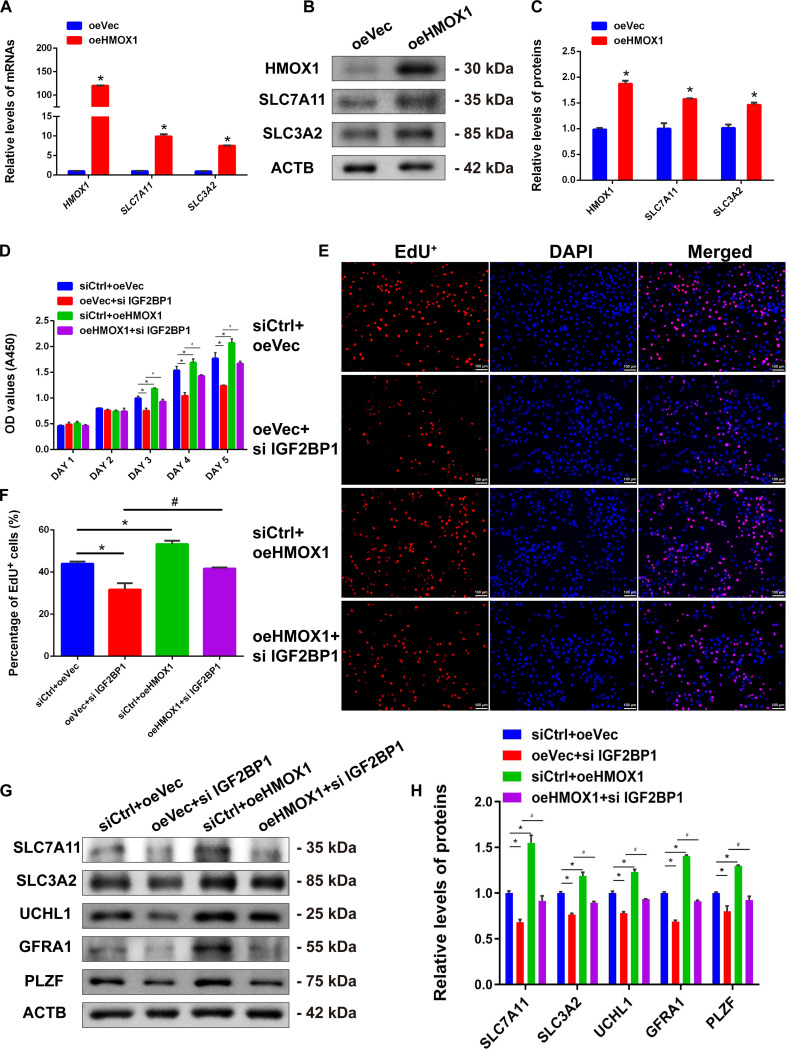
HMOX1 overexpression reversed the effect of IGF2BP1 knockdown on human SSCs. (A to C) Changes in mRNA (A) and protein (B and C) expression of HMOX1, SLC7A11, and SLC3A2 in human SSCs after transfection with HMOX1 expression plasmid. (D) CCK-8 assay showed that HMOX1 overexpression reversed the proliferative capacity of human SSCs by IGF2BP1 knockdown. (E and F) EdU incorporation assays demonstrated that HMOX1 overexpression restored DNA synthesis in human SSCs after IGF2BP1 knockdown. (G and H) Western blot indicated that HMOX1 overexpression reversed the decreases in SLC7A11 and SLC3A2 as well as stem cell-related markers UCHL1, GFRA1, and PLZF proteins in human SSCs after IGF2BP1 knockdown. *P < 0.05, with statistically significant differences. Scale bars, 100 μm.

To determine whether HMOX1 mediates IGF2BP1-regulated fate determinations of human SSCs, we constructed an HMOX1 overexpression plasmid. Transfection of the HMOX1 overexpression plasmid significantly enhanced both HMOX1 mRNA and protein levels (Fig. 10A to C) as well as the expression of SLC7A11 and SLC3A2 (Fig. 10A to C), indicating that HMOX1 may influence human SSC fate determinations by regulating SLC7A11/SLC3A2 expression. Subsequently, we performed rescue experiments for HMOX1 and IGF2BP1 in human SSCs. Following IGF2BP1 knockdown, HMOX1 overexpression was used to assess whether it could reverse the decreased proliferation of human SSCs caused by IGF2BP1 silencing. CCK-8 assays showed that HMOX1 overexpression promoted human SSC proliferation and counteracted the proliferative inhibition induced by IGF2BP1 knockdown (Fig. 10D), with consistent results from EdU incorporation assays (Fig. 10E and F). These results indicate that HMOX1 ameliorates the effect of IGF2BP1 knockdown on human SSC proliferation and DNA synthesis.

Furthermore, HMOX1 overexpression restored the reduced expression of SLC7A11 and SLC3A2 in human SSCs caused by IGF2BP1 knockdown (Fig. 10G and H), suggesting that the influence of IGF2BP1 on SLC7A11/SLC3A2 and ferroptosis is dependent upon HMOX1 regulation. Additionally, IGF2BP1 knockdown decreased the expression levels of stemness-related proteins, including UCHL1, GFRA1, and PLZF, which was rescued by HMOX1 overexpression (Fig. 10G and H). Considered together, these data indicate that IGF2BP1 maintains human SSC stemness and inhibits ferroptosis via the HMOX1 pathway.

### IGF2BP1 regulates the ferroptosis of human SSCs by inhibiting the expression of system Xc-related proteins

Ferroptosis is an iron-dependent form of regulated cell death [26], and it is primarily triggered through 3 pathways, including the system Xc^−^/GPX4 pathway [27], the lipid metabolism pathway [28], and the iron metabolism pathway [29]. We demonstrated that knockdown of IGF2BP1 and/or HMOX1 significantly reduced the expression levels of SLC7A11 and SLC3A2 (Figs. 4A and B and 8I and J). SLC7A11 and SLC3A2 are components of the system Xc^−^ amino acid transporter, which mediates the exchange of extracellular cystine for intracellular glutamate [30]. This system imports cystine into cells, a precursor for GSH synthesis. GSH, in turn, scavenges ROS to maintain cellular redox homeostasis [31]. Dysfunction of system Xc^−^ leads to the reduced GSH production, the increased lipid peroxidation, and subsequent ferroptosis [32,33]. Our immunocytochemistry revealed that IGF2BP1 and HMOX1 knockdown significantly decreased SLC7A11 expression on the cytoplasm of human SSCs (Fig. 9F). Consistently, intracellular GSH levels were markedly reduced by IGF2BP1 and HMOX1 knockdown (Fig. 9G). Collectively, these findings indicate that the IGF2BP1–HMOX1 axis suppresses the ferroptosis in human SSCs by maintaining system Xc^−^ function and GSH-dependent antioxidant defenses.

### The correlation between IGF2BP1 and the occurrence of NOA

**Fig. 11.**
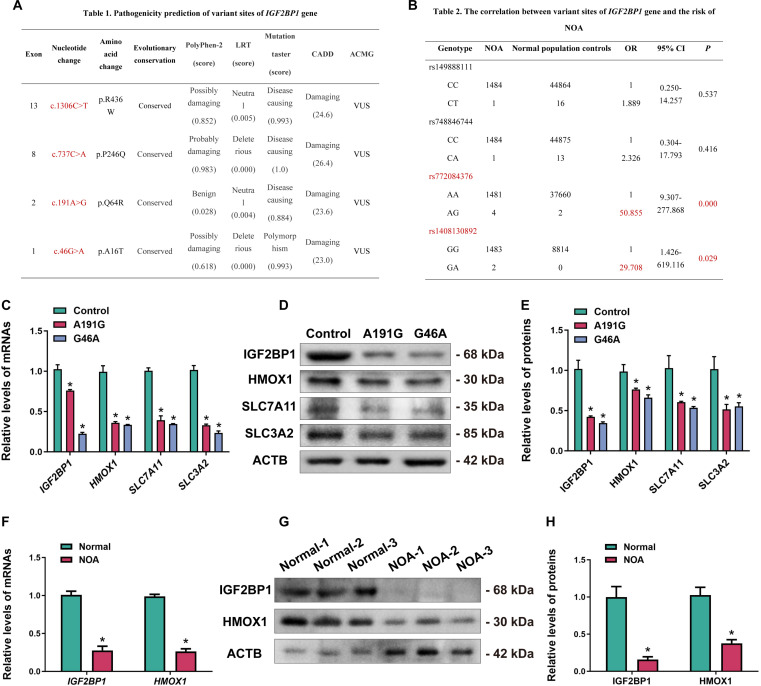
*IGF2BP1* variants of NOA patients and expression comparison of IGF2BP1 and HMOX1 in testicular tissues between normal individuals and NOA patients. (A) Pathogenic prediction of *IGF2BP1* gene variants of NOA patients. (B) Analysis of genotype frequencies of *IGF2BP1* gene variant sites and NOA risk. (C to E) Real-time PCR and Western blot showed that *IGF2BP1* A191G and G46A mutations affected the expression of IGF2BP1, HMOX1, SLC7A11, and SLC3A2 mRNAs and proteins in human SSCs. (F to H) Expression levels of IGF2BP1 and HMOX1 transcripts and proteins in normal testicular tissues and NOA testicular tissues. *P < 0.05, with statistically significant differences.

In recent years, with the development of high-throughput sequencing technologies, an increasing number of reproduction-related genes have been identified as potentially susceptible genes for NOA, e.g., *SYCP2* and *NR5A1* [34–36]. Mutations in these genes may result in spermatogenesis arrest. To evaluate whether *IGF2BP1* gene variations are associated with the occurrence of NOA, we analyzed variations in the *IGF2BP1* gene using whole-exon sequencing (WES) data from 1,485 patients with NOA. We found that 63 cases (4.2%, 63/1485) carried *IGF2BP1* gene variations, indicating its potential role in the NOA pathogenesis. To evaluate variant pathogenicity, we used pathogenicity prediction software (PolyPhen-2, LRT, MutationTaster) for functional analysis and SiPhy, GERP, and PhyloP for conservation analysis. Four missense mutations with potential pathogenicity were identified in *IGF2BP1* (Fig. 11A). To assess the correlation between *IGF2BP1* variations and NOA risk, we compared allelic frequencies of rs149888111, rs748846744, rs772084376, and rs1408130892 between NOA patients and healthy men with normal spermatogenesis. Notably, rs772084376 (A191G) and rs1408130892 (G46A) showed significant frequency differences (*P* < 0.05) with odds ratios (OR) >1, indicating positive correlation with NOA risk (Fig. 11B). To further investigate the impact of these mutations on human SSCs, we constructed *IGF2BP1* expression plasmids carrying A191G and G46A mutations and transfected them into human SSCs. Real-time PCR and Western blot analysis showed that A191G and G46A mutants significantly reduced *IGF2BP1* mRNA and protein expression, which led to decreases in downstream effectors HMOX1, SLC7A11, and SLC3A2 (Fig. 11C to E). These findings suggest that *IGF2BP1* A191G and G46A mutations adversely affect human SSC fate determinations by decreasing *IGF2BP1* and downstream target expression, which potentially contributes to NOA development. Furthermore, to explore whether the down-regulation of this key downstream target, HMOX1, has a direct genetic link to NOA, we analyzed *HMOX1* variants in WES data. Through pathogenicity prediction of HMOX1 variants based on WES data from 691 NOA patients, we identified that the c.58G>A, c.246G>T, and c.836C>T variants were likely pathogenic (Fig. S7A). Subsequent analysis indicated that all 3 variants were positively associated with NOA occurrence (Fig. S7B). This independent genetic association suggests that *HMOX1* gene may serve as a potential risk predictor and clinical therapeutic target for NOA patients.

MHA database analysis revealed that *IGF2BP1* is down-regulated in testicular tissues of NOA patients. Pathogenicity prediction and risk association analysis further indicated that *IGF2BP1* variants are pathogenic, suggesting their critical role in spermatogenesis disorder. To demonstrate these findings, we collected testicular tissues from normal men controls and NOA patients, and we found that both *IGF2BP1* and *HMOX1* mRNA expression levels were significantly decreased in NOA tissues compared to normal controls (Fig. 11F). Western blot analysis revealed lower levels of IGF2BP1 and HMOX1 proteins in the testes of NOA patients than those of control normal men (Fig. 11G and H). Taken together, these results indicate that low expression levels of IGF2BP1 and HMOX1 in NOA testicular tissues are associated with the impaired spermatogenesis and suggest IGF2BP1 and HMOX1 to be potential biomarkers for the diagnosis and treatment of NOA patients.

## Discussion

Human SSCs are the only stem cells within the seminiferous tubules that are responsible for maintaining the continuity and stability of normal spermatogenesis [37]. Therefore, the in-depth studies of the molecular mechanisms underlying fate determinations of human SSCs are of particular importance for understanding the complex processes of spermatogenesis and pathogenesis of male infertility. Here, we revealed the critical regulatory role of IGF2BP1 in the proliferation, stemness maintenance, and novel cell death of human SSCs, and importantly, we explored a new mechanism by which IGF2BP1 influences human SSC fate decisions through HMOX1 m6A modification. Knockdown of IGF2BP1 significantly decreased the proliferation and DNA synthesis capacity of human SSCs, and IGF2BP1 silencing resulted in decreases in expression levels of SSC marker proteins GFRA1, PLZF, and UCHL1 as well as the up-regulation of the differentiation-related protein c-KIT, which implies that IGF2BP1 plays a crucial role in maintaining stemness and self-renewal of SSCs. Xenotransplantation of human SSCs into recipient mouse testes further demonstrated that IGF2BP1 regulates human SSC self-renewal in vivo. When analyzing the mechanisms underlying IGF2BP1-mediated regulation of human SSC death, we found that IGF2BP1 was primarily involved in the regulation of autophagy and ferroptosis rather than apoptosis or pyroptosis. Autophagy, a process of cellular self-degradation and recycling of intracellular components, maintains cell homeostasis by removing damaged organelles and protein aggregates [38] and exerts protective effect against metabolic stress and oxidative damage [39]. We observed that IGF2BP1 knockdown significantly promoted the ferroptosis of human SSCs. Notably, the pro-ferroptosis effect of IGF2BP1 may be linked to its regulation of autophagy. Emerging evidence has indicated the dual role of autophagy in ferroptosis: It can promote ferroptosis by degrading ferritin to release free iron and causing iron overload [40,41], while mitophagy inhibits ferroptosis by reducing mitochondrial ROS and limiting lipid peroxidation [42,43]. For instance, a novel nanozyme D/P@ZUCO exacerbates ferroptosis by inhibiting autophagy, thereby exerting antitumor effect [44]. Consistently, our results suggest that autophagy inhibition by IGF2BP1 silencing promotes the ferroptosis of human SSCs. We thus propose that IGF2BP1 regulates human SSC ferroptosis by modulating autophagy, which plays a protective role in maintaining stemness of human SSCs. Importantly, the specific autophagy dysregulation likely occurs at the level of autophagic flux. The impairment of complete autophagic flow might be the critical event that disrupts cellular homeostasis and predisposes SSCs to ferroptosis. It would be interesting to determine whether the stimulation of autophagic flux can rescue the ferroptosis caused by IGF2BP1 silencing in human SSCs.

We further explored the specific molecular mechanisms by which IGF2BP1 regulates the fate determinations of human SSCs. For the first time, we identified HMOX1 as a downstream target of IGF2BP1 in governing SSC fate determinations. We observed an interaction between IGF2BP1 protein and *HMOX1* mRNA in human SSCs, and interestingly, we revealed that IGF2BP1 modulated *HMOX1* mRNA stability through m6A methylation, thereby regulating human SSC proliferation and stemness. HMOX1 plays an essential role in iron homeostasis by inhibiting ferroptosis in human SSCs. To validate the regulatory role between IGF2BP1 and HMOX1, we knocked down IGF2BP1 and then overexpressed HMOX1, and notably, we found that human SSC proliferation capacity and stemness marker expression were restored, with significantly reduced ferroptosis levels. This finding further demonstrated the pivotal role of the IGF2BP1–HMOX1 signaling axis in regulating human SSC fate determinations, highlighting HMOX1 as a central mediator in maintaining the function and stability of human SSCs. As an essential component of the cellular antioxidant defense system, HMOX1 exhibits a dual role in mediating ferroptosis depending upon the context. For example, HMOX1 induces ferroptosis by inhibiting GPX4 expression in liver and gallbladder cancer [45]. Meanwhile, in endometrial stromal cells, METTL3 down-regulation stabilizes *HMOX1* mRNA to suppress ferroptosis, and HMOX1 overexpression reduces oxidative damage to inhibit ferroptosis [46]. This underscores HMOX1’s context-dependent function in different cell types. Although HMOX1 has been known to participate in the oxidative stress response and iron metabolism, its specific role in maintaining stemness of adult stem cells remains unclear. Our study provides new insights into HMOX1-mediated regulation of stemness in human SSCs, and it offers novel research directions for exploring stem cell function and mechanisms. Our further exploration of the molecular mechanisms by which IGF2BP1 and HMOX1 regulate human SSC ferroptosis revealed that IGF2BP1 and HMOX1 silencing led to decreased expression levels of SLC7A11 and SLC3A2 as well as membrane localization of SLC7A11 in human SSCs. These data suggest that IGF2BP1 and HMOX1 knockdown may regulate intracellular GSH synthesis by affecting the expression and localization of SLC7A11 and SLC3A2 in system Xc^−^, thereby inducing human SSC ferroptosis.

With the development of sequencing technologies (e.g., WES, single-cell RNA-seq, single cell transcriptomes, and proteomics), more than 100 mutated genes related to NOA have been identified by peers and us [47]. It has been reported that MutS homologs 4 and 5 (MSH4, MSH5) play critical roles in meiosis [48]. WES analysis has revealed variants in the *MSH5* and *MSH4* genes in NOA patients, both of which lead to spermatocyte arrest [49,50]. The construction of a homozygous Msh5-mutated male mouse model demonstrates complete infertility, consistent with the phenotypes of patients with NOA. Overexpression of Msh5 in mouse seminiferous tubules restores the limited mature sperm production [49], which provides a new insight into clinical NOA treatment and lays the foundation for precision medicine and personalized therapy. Additionally, X-linked *RBBP7* pathogenic variants cause spermatogenic arrest. *RBBP7* gene variants have been identified in NOA patients, and cell and animal model experiments have shown that Rbbp7-knockout mice exhibit meiotic arrest and increased apoptosis in spermatogenic cells [51]. Our study analyzed WES data and found that variants in the *IGF2BP1* and *HOMX1* genes are positively associated with NOA, which leads to reduced protein levels of IGF2BP1, HMOX1, SLC7A11, and SLC3A2. However, the precise mechanisms by which *IGF2BP1* mutations affect the expression of its downstream targets remain unclear. Future investigations will combine the in silico prediction with functional assays to elucidate the networks by IGF2BP1 and its regulatory molecules. Notably, *IGF2BP1* and its protein expression was significantly down-regulated in testicular tissues of NOA patients, indicating that its functional loss may contribute to abnormal spermatogenesis. Future in vivo experiments using gene-knockout animals will further explore the molecular mechanisms of IGF2BP1 and HMOX1 in NOA pathogenesis and evaluate their feasibility as therapeutic biomarkers.

This study reveals that IGF2BP1 maintains *HMOX1* mRNA stability and system Xc^−^ expression in an m6A-dependent manner, thereby preserving the stemness of human SSCs and inhibiting ferroptosis. Future research could further explore the clinical potential of the IGF2BP1–HMOX1 axis, e.g., developing targeted drugs or utilizing gene editing technologies for precise gene regulation to restore human SSC function, intervene in abnormal spermatogenesis, and offer new therapeutic options for NOA patients.

## Conclusion

**Fig. 12.**
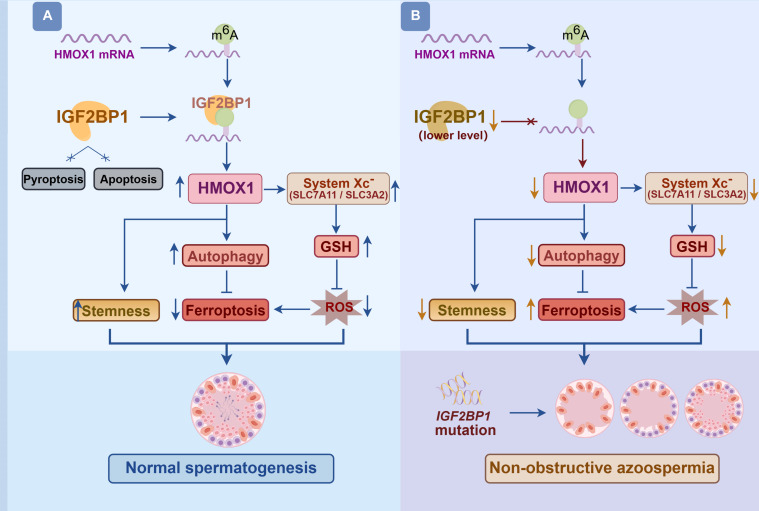
The functions and mechanisms of IGF2BP1 in regulating fate determinations of human SSCs and its dysfunction with male infertility. (A) IGF2BP1 regulates the proliferation, stemness maintenance, ferroptosis, and autophagy by stabilizing *HMOX1* mRNA in an m6A-dependent manner and modulating system Xc^−^ expression to maintain normal spermatogenesis. (B) *IGF2BP1* SNPs and lower levels of IGF2BP1 are closely related to the risk of male infertility.

In summary, we systematically elucidate the critical role and mechanism of IGF2BP1 in regulating the fate decisions of human SSCs. Knockdown of IGF2BP1 inhibits human SSC proliferation, disrupts stemness maintenance, and induces ferroptosis by reducing autophagy (Fig. 12A). Mechanistically, IGF2BP1 stabilizes *HMOX1* mRNA in an m6A-dependent manner and regulates cell fate determinations of human SSCs by modulating system Xc^−^ expression (Fig. 12A). Clinical correlation analysis reveals that the IGF2BP1 and HMOX1 gene and protein are down-regulated in NOA, and importantly, *IGF2BP1* gene variants are positively associated with the risk of NOA (Fig. 12B). As such, this study provides novel insights into understanding the molecular mechanisms underlying fate decision of human SSCs and the etiology of male infertility and identifies new targets for therapeutic intervention of this serious disease.

## Materials and Methods

### The acquirement of human testicular tissues

The testicular tissues from patients with NOA and OA used in this study were obtained from Hunan Cancer Hospital. All patients were fully informed of the sample usage, research objectives, and potential implications, and they voluntarily provided the written informed consent. This study was approved by the Ethics Committee of Hunan Normal University (no. 2021227).

### Cell culture of human SSCs and siRNA or plasmid transfection

The human SSC line was cultured in Dulbecco’s modified Eagle’s medium (DMEM)/F-12 supplemented with 10% fetal bovine serum (FBS; Gibco, USA) and 1% penicillin–streptomycin (PS; Gibco, USA). Cells were cultured in a humidified atmosphere of 5% CO₂ incubator at 34 °C.

Human SSCs were seeded at 50% confluence in 6- or 96-well plates 24 h before transfection. For siRNA transfection, transfection complexes were prepared by diluting 3.75 μl of Lipofectamine 3000 (Invitrogen, USA) and 20 μM siRNA separately in 125 μl of Opti-MEM, and they were incubated for 5 min and then combined with a 1:1 (v/v) ratio. The mixture was incubated with human SSCs for an additional 20 min. The siRNA sequences for IGF2BP1 and HMOX1 oligonucleotides were listed in Table S1. Following a 4- to 6-h transfection of siRNAs, the medium was replaced with fresh complete medium. For plasmid transfection, the P3000 reagent was employed.

### Immunocytochemistry and immunohistochemistry

For immunocytochemistry, human SSCs were fixed with 4% paraformaldehyde (PFA) for 15 min at room temperature and permeabilized with 0.1% Triton X-100 for 10 min. After 3 washes with phosphate-buffered saline (PBS), cells were blocked with immunostaining blocking buffer in a humidified chamber for 2 h at room temperature. Without washing, primary antibodies (diluted in blocking buffer) were directly added to cells and incubated overnight at 4 °C in the dark. Following 3 times of PBS washes, corresponding fluorescence-conjugated secondary antibodies were applied and incubated for 1 h at room temperature in the dark. Cells were then washed 3 times with PBS and mounted using antifade mounting medium containing 4′,6-diamidino-2-phenylindole (DAPI), and they were coverslipped. Fluorescent images were acquired under a fluorescence microscope (Leica DM3000LED, Germany) or a confocal microscope (Leica TCS SP8 SR, Germany).

For immunohistochemistry, the paraffin sections of testicular tissues were placed on a slide warmer and baked at 60 °C for 1 h. Then, the sections were dehydrated using a gradient of alcohols and immersed in 1× sodium citrate antigen retrieval buffer. The buffer was heated in a microwave at high power for 20 min. After the retrieval solution cooled to room temperature, the immunostaining permeabilization solution was applied to the sections that were then incubated at room temperature for 30 min. The subsequent blocking, primary and secondary antibodies’ incubation, and mounting steps were similar to those in immunocytochemistry. Antibodies used in both immunocytochemistry and immunohistochemistry were shown in Table S2.

### Total RNA isolation, RT-PCR, and real-time PCR of human SSCs

Total RNA isolation was performed using Trizol reagent (Vazyme, China) pursuant to the manufacturer’s instructions. The amount and integrity of the extracted RNA were evaluated by measuring absorbance ratios at 260/280 and 260/230 nm with a NanoDrop One spectrophotometer (Thermo Fisher Scientific, USA). Subsequently, the Evo M-MLV RT Premix (AG11706, Accurate Biology, China) was used to reverse-transcribe (RT) the total RNA into complementary DNA (cDNA), which served as templates for subsequent RT-PCR and quantitative real-time PCR analyses.

RT-PCR was performed using 2×Taq Master Mix (Dye Plus) (Vazyme, China) according to the manufacturer’s protocol. The PCR conditions were as follows: initial denaturation at 95 °C for 5 min, followed by 35 cycles of PCR: 95 °C for 30 s, 58 °C for 30 s, and 72 °C for 5 min. PCR products were separated on 2% agarose gels stained with GoldView, and their images were captured using a Gel Documentation System (ChampGel 5000, Sinsage, China).

Quantitative real-time PCR was carried out using the 2× SYBR Green Premix Pro Taq HS qPCR Kit (AGbio, China) in a 20-μl reaction volume, with amplification and detection performed on the Bio-Rad CFX system. Relative gene expression levels were calculated by normalizing to *ACTB* expression using the 2^−ΔΔCT^ method. The primer sequences were shown in Table S3.

### Western blots and Co-IP of human SSCs

Human SSCs were lysed on ice for 30 min using radioimmunoprecipitation assay (RIPA) buffer supplemented with 1 mM phenylmethylsulfonyl fluoride (PMSF), with vortexing every 5 min to facilitate cell disruption. Following centrifugation at 12,000 rpm for 15 min at 4 °C, the supernatant was carefully transferred to a fresh tube. An appropriate volume of sodium dodecyl sulfate–polyacrylamide gel electrophoresis (SDS-PAGE) loading buffer was added to the protein samples, which were then denatured at 99 °C for 10 min. Proteins samples were stored at −20 °C until further analysis.

Following separation by 10% SDS-PAGE, proteins were transferred onto 0.45-μm polyvinylidene fluoride (PVDF) membranes (Millipore, USA). Membranes were blocked with 5% nonfat milk in tris-buffered saline with Tween 20 (TBST) for 1 h at room temperature. Primary antibodies (Table S4) were applied overnight at 4 °C. After washing, horseradish peroxidase (HRP)-conjugated secondary antibodies were incubated for 1 h at room temperature. Protein bands were detected using enhanced chemiluminescence (ECL) reagents (Beyotime, China), and blots were imaged with MiniChemi610 (Sinsage, China). Densitometric analysis of protein blots was performed using ImageJ software.

For Co-IP, human SSCs were lysed in binding/washing buffer, and the supernatant was collected after centrifugation. The supernatant was incubated overnight at 4 °C with specific IGF2BP1 or HMOX1 antibodies against the target protein to form antigen–antibody complexes. The next day, Protein A/G magnetic beads were added, and the mixture was rotated at room temperature for 2 h to capture the complexes. Following multiple washes, the complexes were eluted with SDS-PAGE loading buffer and subjected to Western blot analysis to detect the target protein and its interacting proteins. Antibodies used for Co-IP and Western blot were listed in Table S4.

### CCK-8 assays of human SSCs

Human SSCs were plated into 96-well plates and subjected to transfection with IGF2BP1 or HMOX1 siRNAs, control siRNA, or IGF2BP1 overexpressing plasmids. CCK-8 assays (APExBIO, China) were conducted to assess cell viability at 24, 48, 72, 96, and 120 h post-transfection of siRNAs. According to the manufacturer’s protocol, 10 μl of CCK-8 reagent was added to each well containing 90 μl of DMEM/F-12 medium, followed by incubation at 34 °C for 4 h. Absorbance values at 450 nm were measured using a microplate reader (Synergy 2, Biotek, USA) to determine cell proliferation.

### EdU incorporation assays of human SSCs

Human SSCs were labeled with 10 μM EdU A (Ribobio, China) in medium and incubated at 34 °C with 5% CO₂ incubator for 16 h. Subsequently, cells were fixed with 4% PFA for 15 min at room temperature. After fixation, each well received 2 mg/ml glycine and shaken at room temperature for 5 min, followed by treatment with 0.5% Triton X-100 for 10 min. Apollo staining solution was then prepared per the kit instructions, and it was added to wells of cells and incubated in the dark for 30 min before removal. Cells were washed 3 times with 0.5% Triton X-100 in PBS for 10 min each. Cell nuclei were stained with DAPI, and the percentage of EdU-positive cells was calculated by counting at least 1,000 cells in the acquired images under a fluorescence microscope (Leica).

### Lipid ROS assay of human SSCs

The 2′,7′-dichlorodihydrofluorescein diacetate (DCFH-DA) probe (Dojindo, Japan) was diluted to a final concentration of 10 μM with serum-free medium. At 48 h post-cell transfection of siRNAs, the culture medium was discarded, and the DCFH-DA solution was added to the medium. The cells were incubated at 34 °C for 30 min and gently shaken every 10 min to ensure that the probe evenly entered the cells. After incubation, the staining solution was removed, and the cells were washed 3 times with PBS to eliminate the unincorporated probes. Finally, the cellular fluorescence signals were observed and imaged under an inverted fluorescence microscope (Leica DMi8, Germany).

### FerroOrange imaging assay of human SSCs

Fe^2+^ levels in human SSCs were determined using the commercially available FerroOrange (Dojindo, Japan) according to the manufacturer’s instructions. Briefly, after siRNA treatment, human SSCs were incubated with 1 μM FerroOrange dissolved in HBSS (Hanks’ balanced salt solution) at 34 °C in a 5% CO₂ incubator for 30 min. During the incubation period, the cell culture plate was gently shaken every 10 min to ensure even distribution of the probe. After the incubation was completed, the cells were washed 3 times with prewarmed HBSS to remove unbound FerroOrange. Finally, the cellular fluorescence signals were observed and imaged under an inverted fluorescence microscope (Leica DMi8).

### Measurement of GSH and MDA levels in human SSCs

MDA in human SSCs was determined using the commercially available MDA Assay Kit (Elabscience, China) pursuant to the manufacturer’s instructions. In total, 100 μl of the cell sample was mixed with 200 μl of MDA reagent and vortexed thoroughly. The mixture was incubated in a 95 °C water bath for 60 min to promote the reaction between MDA and thiobarbituric acid (TBA) to produce a red compound. After the reaction, the samples were rapidly cooled in an ice bath for 10 min and then centrifuged at 12,000 rpm for 10 min at 4 °C. The optical density (OD) of the supernatant was measured at 532 nm using a microplate reader (Synergy 2, Biotek, USA).

For GSH determination in human SSCs, a specific GSH assay kit (Sangon Biotech, China) was employed. Totally, 50 μl of the collected supernatant was added to a 96-well plate, followed by the addition of 150 μl of the GSH-specific reaction mixture provided in the kit. The plate was incubated at room temperature for 20 min in the dark. Subsequently, the absorbance was measured at 412 nm using a microplate reader (Synergy 2, Biotek, USA).

### RNA-seq of human SSCs

RNA-seq and analysis of human SSCs were conducted by MAJORBIO (Shanghai, China). Total RNA was extracted from human SSCs using Trizol reagent. Only high-quality RNA samples [OD_260/280_ = 1.8 to 2.1, OD_260/230_ ≥ 2.0, RQN (RNA quality number) ≥ 6.5] were applied to construct the sequencing libraries with the Illumina NovaSeq Reagent Kit. The mRNA was purified, fragmented, reverse-transcribed to cDNA, and underwent end-repair, A-tailing, adapter ligation, and PCR enrichment. The libraries were sequenced using the NovaSeq X Plus sequencer (2 × 150-base pair read length). Raw reads were processed to obtain clean reads, which were aligned to the human reference genome (GRCh38) using HISAT2. DEGs were analyzed with DESeq2. Genes with |log₂FC| ≥ 0.585 and *P* < 0.05 were defined as the DEGs. The GO functional enrichment and KEGG pathway analysis were carried out by Goatools and Python scipy software, respectively.

### Xenotransplantation of human SSCs into mouse seminiferous tubules

To establish a recipient animal model with no male germ cell, mice were injected intraperitoneally with 40 mg/kg of busulfan. Four weeks after busulfan treatment, H&E staining was performed on mouse testis sections to observe spermatogenesis. The model was considered to be successfully established when only Sertoli cells were present in the lumen without male germ cells.

For the xenotransplantation of human SSCs, the abdominal skin of mice was incised with presterilized surgical instruments to expose the abdominal cavity. One testis was gently lifted out using nontoothed forceps and placed on a sterile pad. Under a stereomicroscope (Olympus, Japan), the efferent ductules were isolated with fine forceps, and then 1 × 10^6^ human SSCs were transplanted. For 3 consecutive days post-surgery, each mouse was administered with 1 ml of 4% PS to prevent postoperative infection. Two months after human SSC transplantation, the testes were harvested under anesthesia, and they were embedded, sectioned, and subjected to immunohistochemical staining.

### RNA dot blotting

The extracted RNA was serially diluted and denatured by heating in a 95 °C metal bath for 3 min. The denatured samples were then spotted onto the nylon membranes and cross-linked under an ultraviolet lamp for 30 min. After cross-linking, the membranes were incubated in blocking solution at room temperature for 1 h, followed by incubation with m6A primary antibody on a shaker at 4 °C overnight. The next day, the membranes were incubated with secondary antibody at room temperature for 1 h. Following extensive washing to remove unbound secondary antibody, the membranes were detected using ECL solution, and images were taken with a chemiluminescent imaging system.

### The m6A RNA methylation quantification

The overall m6A modification levels in human SSCs were determined using the EpiQuik m6A RNA Methylation Quantification Kit (Epigentek, China). Initially, total RNA was extracted from human SSCs using Trizol. Subsequently, the extracted RNA was firmly immobilized within the wells of a microplate by applying the high-binding solution provided in the kit. The capture and detection antibodies included in the kit were used to recognize m6A specifically. Following signal enhancement, colorimetric quantification was carried out using a microplate reader at 450 nm.

### MeRIP-qPCR and RIP-qPCR

Total RNA was isolated using the Trizol method and kept on ice. RNA was fragmented using an ultrasonic disruptor (90 W, 5 s each time, 5-s interval, for 15 times). The m6A antibody was bound to magnetic beads using 50 μl of Protein A/G magnetic beads, 5 μg of m6A antibody, and incubating the mixture at room temperature for 1 h. Then, 5 μg of fragmented RNA and ribonuclease (RNase) inhibitor was added to the antibody-bound magnetic beads with the addition of the IP reaction solution. The mixture was incubated overnight at 4 °C with rotation, and the m6A elution solution was added and incubated at 34 °C for 30 min to recover the immunoprecipitated RNA. Subsequently, RNA was extracted again using Trizol, and real-time PCR was conducted to compare the changes in m6A methylation modification on *HMOX1* mRNA of human SSCs.

### The mRNA stability assay

To assess the stability of *HMOX1* mRNA, human SSCs were transfected with IGF2BP1 siRNAs. After 48 h of transfection, 5 μg/ml working solution of actinomycin D, an inhibitor of RNA synthesis, was added to each well of human SSCs. The cells were then incubated at 34 °C with 5% CO₂ for 0, 2, 4, 8, and 12 h. Following incubation, RNA was extracted using the Trizol reagent pursuant to the manufacturer’s protocol. Real-time PCR was performed to detect the changes in the relative content of *HMOX1* mRNA to evaluate its stability in human SSCs affected by IGF2BP1 siRNAs.

### IF-FISH

Human SSCs were fixed with 4% PFA for 20 min at room temperature and permeabilized with 0.5% Triton X-100 in PBS for 15 min. Target nucleic acids were denatured by heating at 80 °C for 5 min and followed by overnight hybridization with fluorescently labeled *HMOX1* probes (GenePharma, China) at 37 °C in a specific buffer. After hybridization, cells were washed 3 times with 2× SSC buffer at 42 °C. Nonspecific binding sites were blocked with 5% bovine serum albumin in PBS for 1 h and then incubated with IGF2BP1 antibody (Proteintech, China) overnight at 4 °C. After PBS washes, the cells were incubated with secondary antibodies for 1 h at room temperature. Cellular nuclei were counterstained with DAPI, and cells were mounted with anti-fade medium before being visualized under a confocal or fluorescence microscope.

### Pathogenicity analysis of *IGF2BP1* and *HMOX1* gene variants

We analyzed *IGF2BP1* and *HMOX1* gene variants from WES data and evaluated their population frequencies using databases including gnomAD, 1000 Genomes, and dbSNP. Functional impacts of *IGF2BP1* and *HMOX1* missense mutations were predicted using bioinformatics tools, including PolyPhen-2, MutationTaster, LRT, SIFT, and CADD. Evolutionary importance of *IGF2BP1* and *HMOX1* variant sites was assessed by PhyloP, SiPhy, and GERP. In addition, *IGF2BP1* and *HMOX1* variants were classified into 5 pathogenicity categories based on the 2015 guidelines of the American College of Medical Genetics and Genomics (ACMG), e.g., Pathogenic, Likely Pathogenic, Variant of Uncertain Significance (VUS), Likely Benign, and Benign.

### NOA disease risk analysis for *IGF2BP1* and *HMOX1* SNP

The *IGF2BP1* and *HMOX1* genotypes and allele frequencies of single-nucleotide polymorphism (SNP) loci were statistically analyzed using SPSS 24.0 statistical software. The chi-square test (*χ*^2^ test) was used to compare the distribution of alleles and genotypes of *IGF2BP1* and *HMOX1* between the 2 groups for significant differences (*P* < 0.05 was considered statistically significant). Logistic regression analysis was performed to calculate the OR and 95% confidence interval (CI) to evaluate the impact of different SNPs in *IGF2BP1* and *HMOX1* on the risk of NOA. The mutation frequency of an SNP in the case group was significantly higher than that in the control group, with OR > 1 and the 95% CI not including 1, which indicated that the SNP might be a risk variant for NOA. Conversely, an OR < 1 suggested the potential protective effect.

### Statistical analysis

All experiments were repeated at least 3 times, and data were presented as mean ± standard error of the mean (SEM). Statistical analyses were performed using GraphPad Prism 6.01, and the differences between the control and treatment groups were compared using an independent sample *t* test or one-way analysis of variance (ANOVA). The statistical significance was set at *P* < 0.05, and data were considered statistically significant with *P* < 0.05.

## Data Availability

The data could be available with the consent of the corresponding author.
