# Supplementary material for: IGF2BP1/HMOX1 Mediates Fate Determinations of Human Spermatogonial Stem Cells and Male Infertility via an m6A-Dependent Manner
Source: Research (Wash D C). 2025 Nov 21;8:1005. doi: 10.34133/research.1005 (PMC12635413; doi:10.34133/research.1005)
Supplement: Supplementary 1 — Figs. S1 to S7 Tables S1 to S4 [file research.1005.f1.docx]

**IGF2BP1/HMOX1 Mediates Fate Determinations of Human Spermatogonial Stem Cells and Male Infertility via an m6A Dependent Manner**

Li Du^†^ , Wei Liu^†^, Yinghong Cui, Wei Chen, Zuping He*

**Supplementary Data**

**Figures S1-S7**

**Tables S1-4**

**Figures S1-S7**

**Figure S1**

**
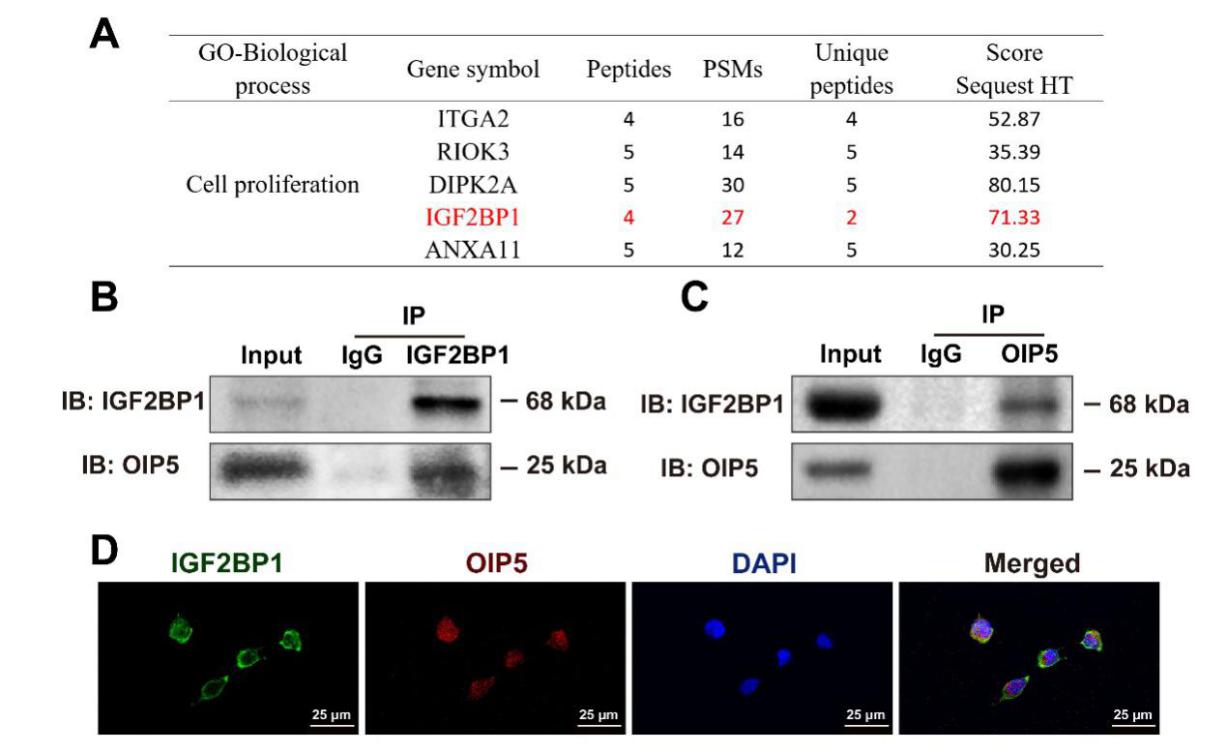
**

**Figure S1. Interaction between IGF2BP1 and OIP5 in human SSCs.** (A) Mass spectrometry analysis indicated an interaction between IGF2BP1 and OIP5 proteins, which was involved in the regulation of cell proliferation pathways. (B-C) Co-IP assay revealed the interaction between IGF2BP1 and OIP5 in human SSCs. (D) Immunocytochemistry showed co-localization of IGF2BP1 and OIP5 in human SSCs. Scale bars, 25 μm.

**Figure S2**

**
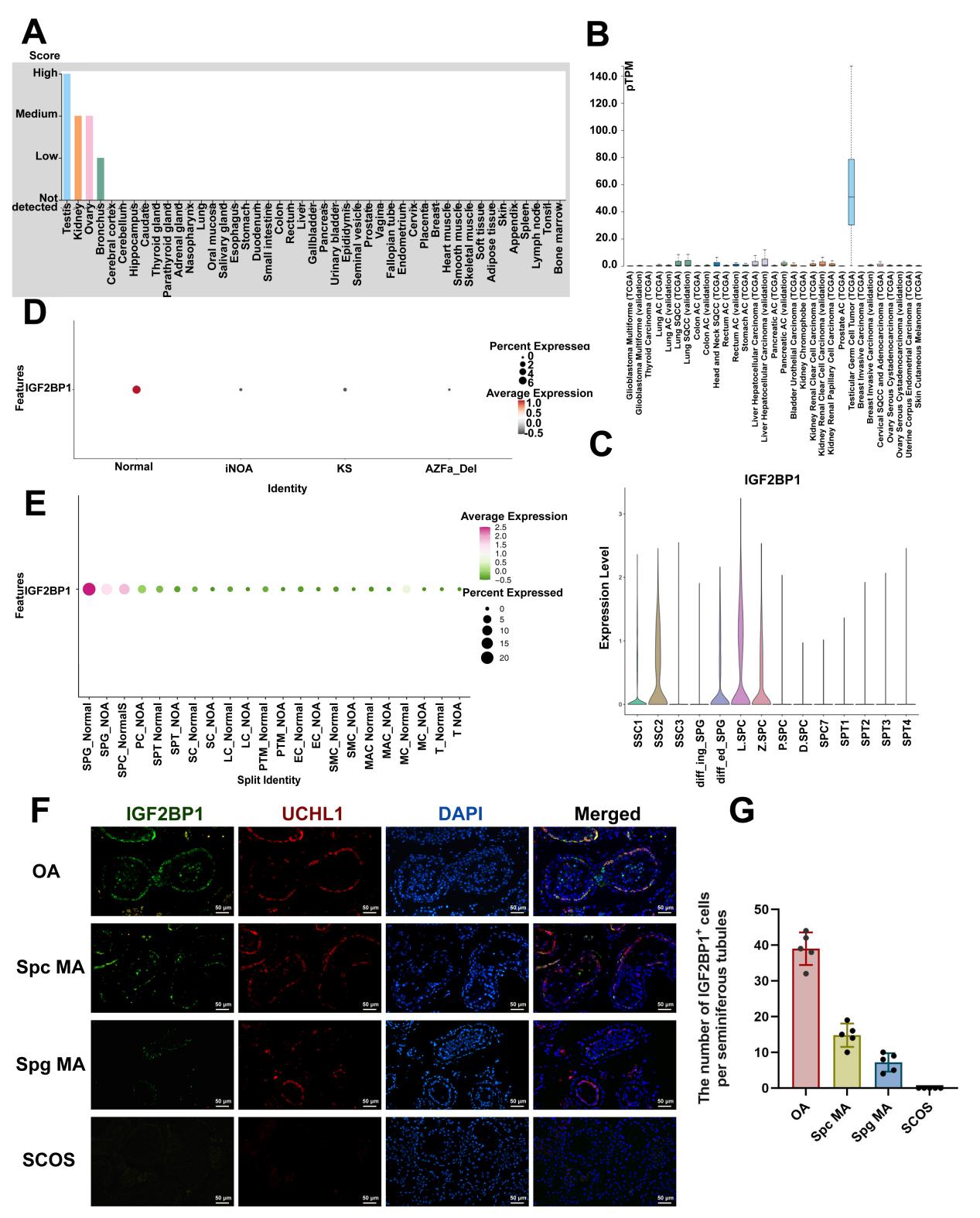
**

**Figure S2. Expression and localization of IGF2BP1 in adult human testicular tissues.** (A) Expression of IGF2BP1 protein in various tissues from the HPA database. (B) Expression levels of IGF2BP1 in different cancer types from TCGA data. (C) Violin plot visualized the expression distribution of IGF2BP1 in all testicular cell types. (D-E) Bubble plot illustrated IGF2BP1 expression in normal testicular tissues and three types of NOA testis tissues. (F-G) Immunohistochemistry revealed the expression levels of IGF2BP1 in the following types of patients: obstructive azoospermia (OA), maturation arrest at spermatogonia (spg MA), maturation arrest at spermatocytes (Spc MA), and Sertoli cell-only syndrome (SCOS). Scale bars, 50 μm.

**Figure S3**

**
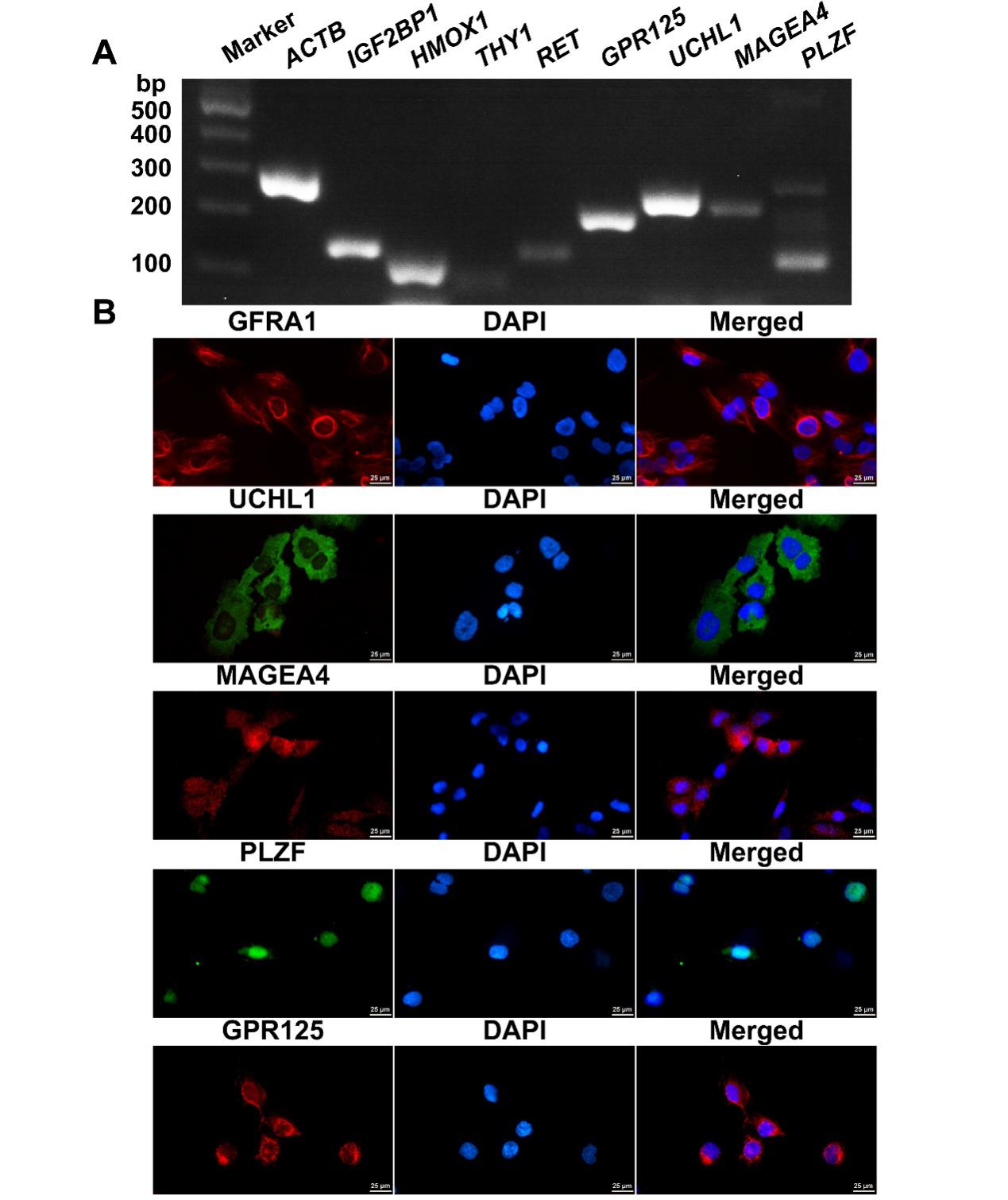
**

**Figure S3. Phenotypic characterization and identification of human SSC line.** (A) Agarose gel electrophoresis showed expression of *THY1*, *RET*, *GPR125*, *UCHL1*, *MAGEA4*, and *PLZF* genes in human SSC line. (B) Immunocytochemistry demonstrates expression of GFRA1, UCHL1, MAGEA4, PLZF, and GPR125 proteins in the human SSC line. Scale bars, 25 μm.

**Figure S4**


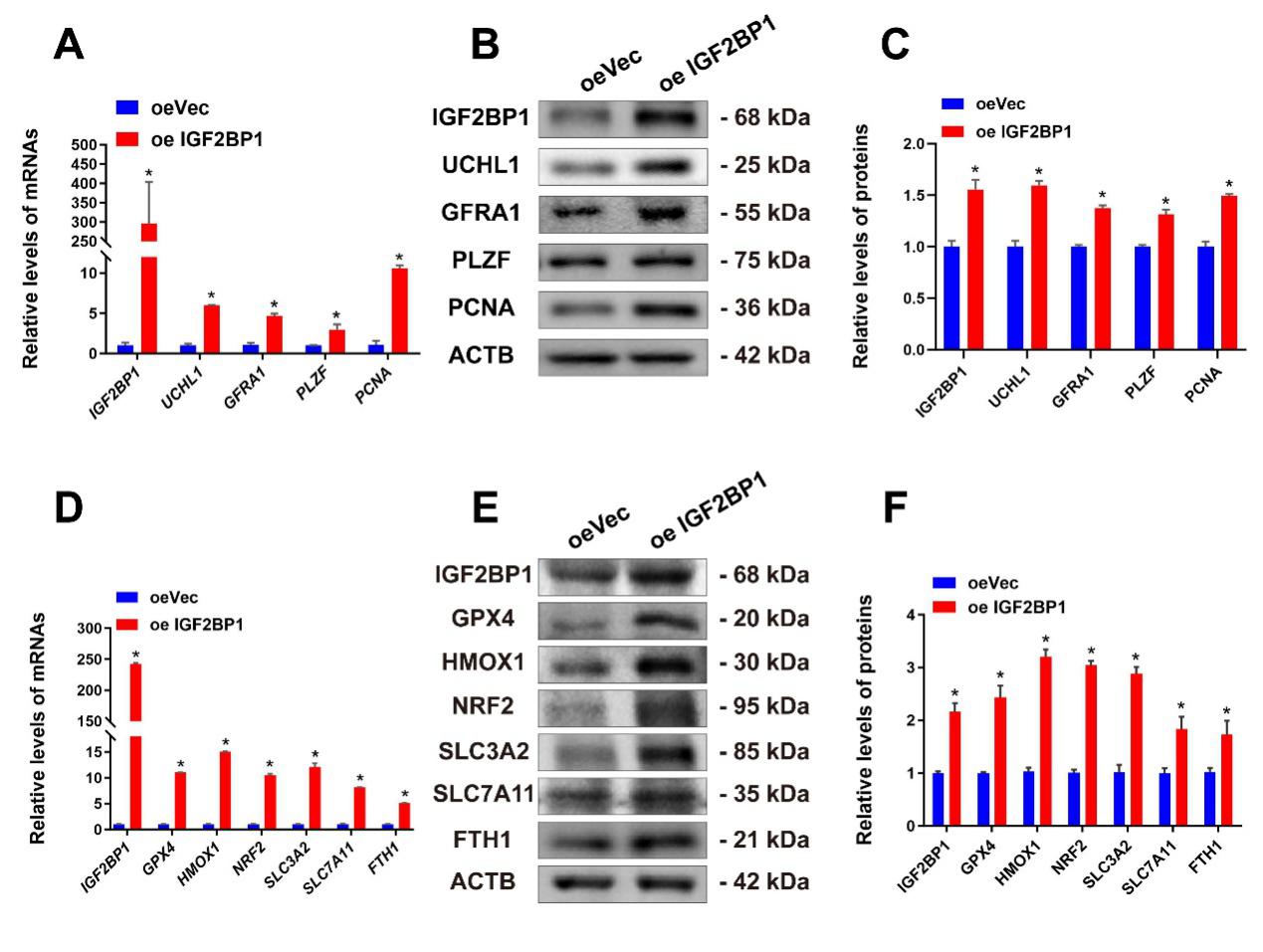


**Figure S4. Effect of IGF2BP1 overexpression on the proliferation, stemness maintenance and ferroptosis in human SSCs.** (A-C) Real-time PCR and Western blots showed changes in the expression of both stemness-related markers (UCHL1, GFRA1 and PLZF) and the proliferation marker PCNA in human SSCs after transfection with IGF2BP1 expression plasmid. (D-F) Real-time PCR and Western blots showed changes in the expression of ferroptosis marker proteins in human SSCs after transfection with IGF2BP1 expression plasmid. * *P* < 0.05 indicated statistically significant differences.

**Figure S5**

**
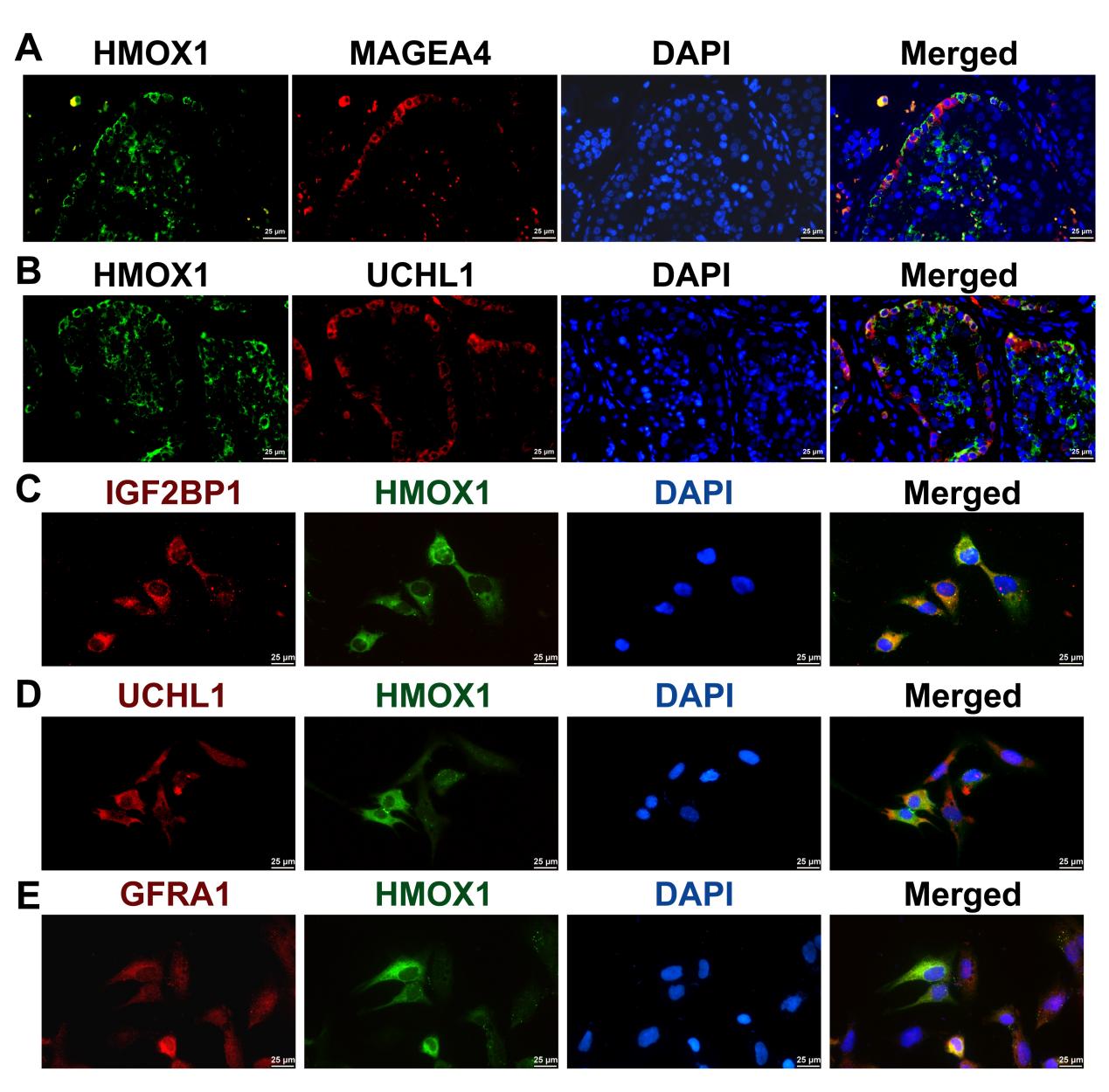
**

**Figure S5. Co-expression of HMOX1 with a number of proteins for human SSCs and spermatogonia in human testicular tissues and human SSC line.** (A) Immunohistochemistry illustrated co-localization of HMOX1 with spermatogonial marker protein MAGEA4 in testicular tissues. (B) Immunohistochemistry demonstrated co-localization of HMOX1 with the SSC marker UCHL1 in testicular tissues. (C) Immunocytochemistry revealed co-localization of HMOX1 and IGF2BP1 in human SSC line. (D) Immunocytochemistry displayed the co-localization of HMOX1 with the SSC marker UCHL1 in human SSC line. (E) Immunocytochemistry showed co-localization of HMOX1 with human SSC marker GFRA1 in human SSC line. Scale bars, 25 μm.

**Figure S6**

**
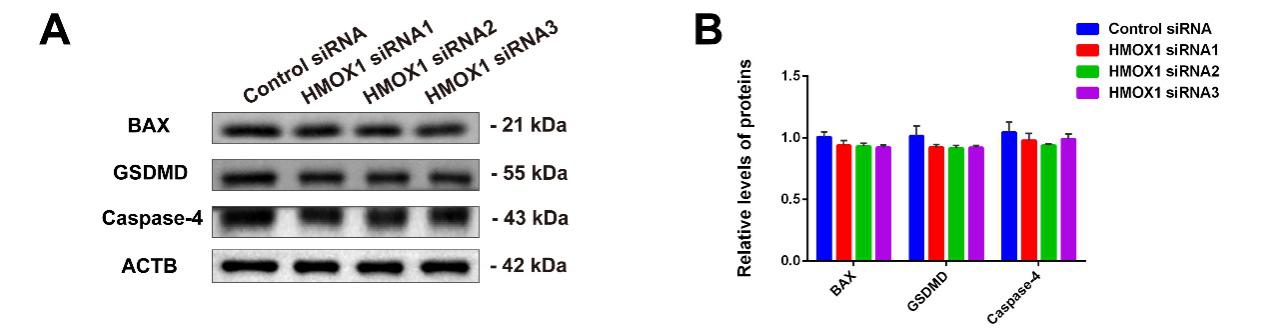
**

**Figure S6. Effect of HMOX1 knockdown on the apoptosis- and pyroptosis-related proteins in human SSCs.** (A-B) Western blots revealed the expression changes of apoptosis- and pyroptosis-related proteins in human SSCs by HMOX1 siRNA1-3. * *P* < 0.05 showed statistically significant differences.

**Figure S7**


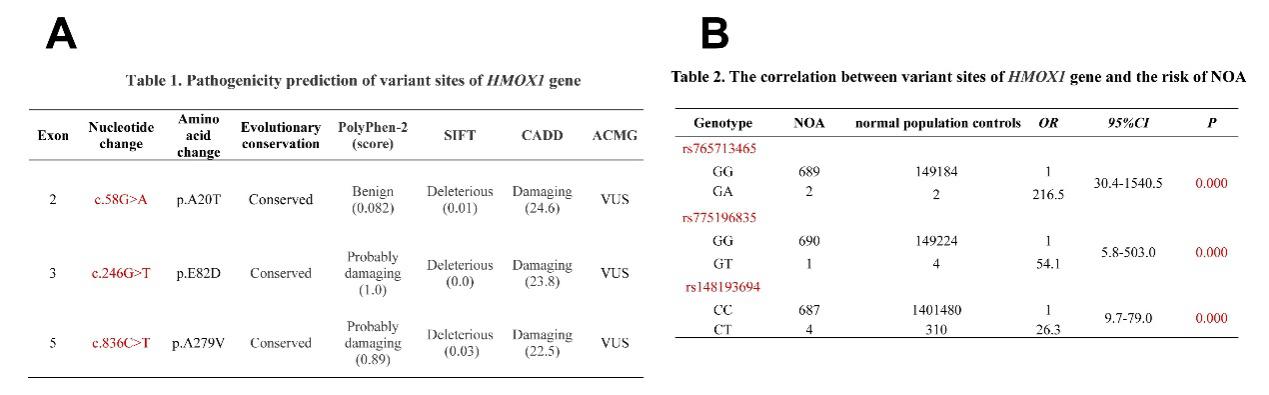


**Figure S7. *HMOX1* variants in NOA patients.** (A) Pathogenic prediction of *HMOX1* gene variants of NOA patients. (B) Analysis of genotype frequencies of *HMOX1* gene variant sites and NOA risk.

**Tables S1-S4**

**Table S1. The siRNA sequences for *IGF2BP1* and *HMOX1* oligonucleotides**

| **SiRNAs** | **Forward sequences (5’-3’)** | **Reverse sequences (5’-3’)** |
| --- | --- | --- |
| *IGF2BP1* siRNA1 | GUUCGUAUGGUUAUCAUCA(dT)(dT) | UGAUGAUAACCAUACGAAC(dT)(dT) |
| *IGF2BP1* siRNA2 | UUGAGUUUGCCAUAGAUUC(dT)(dT) | GAAUCUAUGGCAAACUCAA(dT)(dT) |
| *IGF2BP1* siRNA3 | CCAAAGUUCGUAUGGUUAU(dT)(dT) | AUAACCAUACGAACUUUGG(dT)(dT) |
| *HMOX1* siRNA1 | UUGCGGUGCAGCUCUUCUG(dT)(dT) | CAGAAGAGCUGCACCGCAA(dT)(dT) |
| *HMOX1* siRNA2 | UAGAGCUGCUUGAACUUGG(dT)(dT) | CCAAGUUCAAGCAGCUCUA(dT)(dT) |
| *HMOX1* siRNA3 | UGAACUCAGCAUUCUCUGC(dT)(dT) | GCAGAGAAUGCUGAGUUCA(dT)(dT) |
| Control siRNA | UUCUCCGAACGUGUCACGUTT | ACGUGACACGUUCGGAGAATT |
| Cy3-labeled siRNA | UUCUCCGAACGUGUCACGUTT | ACGUGACACGUUCGGAGAATT |

**Table S2. The detailed information of antibodies for immunocytochemistry and immunohistochemistry**

| **Antibodies** | **Dilutions** | **Companies** | **Catalog numbers** |
| --- | --- | --- | --- |
| IGF2BP1 | 1:100 | Proteintech | 22803-1-AP |
| OIP5 | 1:100 | Proteintech | 12142-1-AP |
| Ki67 | 1:100 | Abcam | ab15580 |
| PCNA | 1:100 | Proteintech | 10205-2-AP |
| HMOX1 | 1:100 | Proteintech | 10701-1-AP |
| SOX9 | 1:20 | Merck | AB5535 |
| GPR125 | 1:50 | Proteintech | 11912-1-AP |
| UCHL1 | 1:100 | Bio-Rad | MCA4750GA |
| GFRA1 | 1:100 | R&D Systems | AF714 |
| CD90 | 1:50 | Proteintech | 66766-1-Ig |
| SLC7A11 | 1:100 | CST | 12691S |
| Anti-Goat Alexa Fluor 555 | 1:1000 | Thermo Scientific | A11058 |
| Anti-Mouse Alexa Fluor 488 | 1:1000 | Thermo Scientific | A10680 |
| Anti-Mouse Alexa Fluor 555 | 1:1000 | Thermo Scientific | A31570 |
| Anti-Rabbit Alexa Fluor 488 | 1:1000 | Thermo Scientific | A32731 |
| Anti-Rabbit Alexa Fluor 555 | 1:1000 | Thermo Scientific | A31572 |
| HumNuc | 1:50 | Abcam | ab191181 |
| SV40 | 1:20 | Santa-Cruz | sc192 |

**Table S3. Gene primers used for RT-PCR and real-time PCR**

| **Genes** | **Species** | **Forward primers (5’-3’)** | **Reverse primers (5’-3’)** |
| --- | --- | --- | --- |
| *ACTB* | Human | CATGTACGTTGCTATCCAGGC | CTCCTTAATGTCACGCACGAT |
| *IGF2BP1* | Human | GCGGCCAGTTCTTGGTCAA | TTGGGCACCGAATGTTCAATC |
| *THY1* | Human | TCACCCATCCAGTACGAGTTC | GGAGCGGTATGTGTGCTCAG |
| *RET* | Human | AAAGTGGCATTGGGCCTCTAC | GCAGGGCATGGACGTACAG |
| *GPR125* | Human | GCGTCATTACGGTCTTTGGAA | ACGGCAATTCAAGCGGAGG |
| *UCHL1* | Human | CCTGTGGCACAATCGGACTTA | CATCTACCCGACATTGGCCTT |
| *MAGEA4* | Human | CTTACCCACTACCATCAGCTTC | TGATGACTCTCTCCAGCATTTC |
| *PLZF* | Human | CCTCAGACGACAATGACACGG | CTCGCTGGAATGCTTCGAGAT |
| *HMOX1* | Human | TGCCAGTGCCACCAAGTTCAAG | TGTTGAGCAGGAACGCAGTCTTG |
| *GPX4* | Human | GAGGCAAGACCGAAGTAAACTAC | CCGAACTGGTTACACGGGAA |
| *NRF2* | Human | TTCCTTCAGCAGCATCCTCTCC | AATCTGTGTTGACTGTGGCATCTG |
| *SLC3A2* | Human | TGAATGAGTTAGAGCCCGAGA | GTCTTCCGCCACCTTGATCTT |
| *SLC7A11* | Human | TCTCCAAAGGAGGTTACCTGC | AGACTCCCCTCAGTAAAGTGAC |
| *FTH1* | Human | CCCCCATTTGTGTGACTTCAT | GCCCGAGGCTTAGCTTTCATT |
| *KEAP1* | Human | CTGGAGGATCATACCAAGCAGG | GGATACCCTCAATGGACACCAC |
| *CPEB4* | Human | ACATCTAGCGCATCGTCTCTT | ACAACAGAGCACCGTTATTAGC |
| *CXCL8* | Human | CACCGGAAGGAACCATCTCA | AGAGCCACGGCCAGCTT |
| *IL1B* | Human | TTGACGGACCCCAAAAGAT | GAAGCTGGATGCTCTCATCTG |
| *RRM2B* | Human | AGAGGCTCGCTGTTTCTATGG | GCAAGGCCCAATCTGCTTTTT |
| *IFNGR1* | Human | TTCCATCTCGGCATACAGCAA | TCTTTGGGTCAGAGTTAAAGCCA |
| *HMGB1* | Human | TATGGCAAAAGCGGACAAGG | CTTCGCAACATCACCAATGGA |
| *CCNG1* | Human | GAGTCTGCACACGATAATGGC | GTGCTTGGGCTGTACCTTCA |
| *HIPK3* | Human | CATATCTACAATCTCGGTACTACAGAGC | GTATCGAATCTGATCATACTCCAAGGCTC |
| *DDIT3* | Human | GGAAACAGAGTGGTCATTCCC | CTGCTTGAGCCGTTCATTCTC |
| *CCNA2* | Human | TTGAACAGTTGGCAGCAC | AGGAGTCGCTCGGAGTC |
| *IL33* | Human | GAGAAATCACGGCAGAATCA | CTTCTTATTTTGCAAGGCGG |

**Table S4. The detailed information of antibodies for Co-IP and Western blots**

| **Antibodies** | **Dilutions** | **Companies** | **Catalog numbers** |
| --- | --- | --- | --- |
| IGF2BP1 | 1:1000 | Proteintech | 22803-1-AP |
| OIP5 | 1:500 | Proteintech | 12142-1-AP |
| PCNA | 1:1000 | Proteintech | 10205-2-AP |
| HMOX1 | 1:1000 | Proteintech | 10701-1-AP |
| GPR125 | 1:1000 | Proteintech | 11912-1-AP |
| UCHL1 | 1:1000 | Bio-Rad | MCA4750GA |
| GFRA1 | 1:1000 | R&D Systems | AF714 |
| CD90 | 1:1000 | Proteintech | 66766-1-Ig |
| PLZF | 1:1000 | Santa-Cruz | sc28319 |
| ACTB | 1:20000 | Proteintech | 66009-1-Ig |
| GPX4 | 1:1000 | CST | [52455](https://www.cellsignal.cn/products/primary-antibodies/gpx4-antibody/52455)S |
| NRF2 | 1:1000 | CST | 12721S |
| SLC3A2 | 1:1000 | CST | 47213S |
| SLC7A11 | 1:1000 | CST | 12691S |
| ACSL4 | 1:1000 | Proteintech | 22401-1-AP |
| FTH1 | 1:1000 | CST | 4393S |
| LC3I/II | 1:1000 | CST | 12741S |
| ATG16L1 | 1:1000 | CST | 8089S |
| Beclin 1 | 1:1000 | CST | 3495S |
| ATG3 | 1:1000 | CST | 3415S |
| Cleaved PARP | 1:1000 | Proteintech | 60555-1-Ig |
| BCL2 | 1:500 | Proteintech | 12789-1-AP |
| BAX | 1:500 | Proteintech | 50599-2-Ig |
| Caspase-3 | 1:1000 | Proteintech | 25128-1-AP |
| Caspase-4 | 1:1000 | Proteintech | 11856-1-AP |
| GSDMD | 1:1000 | Abcam | ab219800 |
| KEAP1 | 1:1000 | CST | 8047S |
| Goat Anti-Mouse HRP | 1:4000 | Beyotime | A0126 |
| Goat Anti-Rabbit HRP | 1:4000 | Beyotime | A0208 |
| IgG | IP: 1μg | Proteintech | B900620 |
| IgG | IP: 1μg | CST | 2729 |
